# Mineralization of *Alvinella* polychaete tubes at hydrothermal vents

**DOI:** 10.1111/gbi.12123

**Published:** 2014-12-30

**Authors:** M N Georgieva, C T S Little, A D Ball, A G Glover

**Affiliations:** 1 School of Earth and Environment, University of LeedsLeeds, UK; 2 Life Sciences Department, The Natural History MuseumLondon, UK; 3 Imaging and Analysis Centre, The Natural History MuseumLondon, UK

## Abstract

Alvinellid polychaete worms form multilayered organic tubes in the hottest and most rapidly growing areas of deep-sea hydrothermal vent chimneys. Over short periods of time, these tubes can become entirely mineralized within this environment. Documenting the nature of this process in terms of the stages of mineralization, as well as the mineral textures and end products that result, is essential for our understanding of the fossilization of polychaetes at hydrothermal vents. Here, we report in detail the full mineralization of *Alvinella* spp. tubes collected from the East Pacific Rise, determined through the use of a wide range of imaging and analytical techniques. We propose a new model for tube mineralization, whereby mineralization begins as templating of tube layer and sublayer surfaces and results in fully mineralized tubes comprised of multiple concentric, colloform, pyrite bands. Silica appeared to preserve organic tube layers in some samples. Fine-scale features such as protein fibres, extracellular polymeric substances and two types of filamentous microbial colonies were also found to be well preserved within a subset of the tubes. The fully mineralized *Alvinella* spp. tubes do not closely resemble known ancient hydrothermal vent tube fossils, corroborating molecular evidence suggesting that the alvinellids are a relatively recent polychaete lineage. We also compare pyrite and silica preservation of organic tissues within hydrothermal vents to soft tissue preservation in sediments and hot springs.

## Introduction

The annelid worms are an ancient lineage of animals dating to at least the earliest Cambrian period, ∼540 Ma (Conway Morris & Peel, 2008; Vinther *et al*., 2011). Over evolutionary time, they have radiated into almost all marine habitats including deep-sea hydrothermal vents. Many vent sites in the Pacific are characterized by spectacular colonies of tube-dwelling polychaetes in the families Siboglinidae and Alvinellidae (Van Dover, 2000). Our understanding of the evolutionary history of these polychaetes and the vent ecosystems more generally is limited by a poor fossil record of soft-bodied organisms. Typically, preservation of soft tissues occurs through early authigenic mineralization (the impregnation and/or replication of an organic structure by minerals) and usually involves the minerals phosphate, carbonate, pyrite or silica (Briggs *et al*., 1991; Akahane *et al*., 2004). Much research has focused on organic tissue mineralization within soft sediments and terrestrial hot springs (e.g. Raff *et al*., 2008; Farrell *et al*., 2013), but mineralization of organic animal and prokaryotic remains within hydrothermal vent environments, which also involves pyrite and silica (Cook & Stakes, 1995; Maginn *et al*., 2002; Boyce *et al*., 2003), is poorly understood. Documenting this process at modern hydrothermal vents is key to understanding taphonomy within this chemically distinct setting and to improving the interpretation of ancient vent fossils.

Worm tube fossils with diverse morphologies are known from vent sites in the geological record back to the early Silurian period, ∼430 million years ago (Little *et al*., 1998, 1999, 2004, 2007; Hilário *et al*., 2011), but little is known about the animals that formed them. Although some have been assigned to extant vent polychaete groups, morphological identifications are not generally consistent with estimates of molecular divergence (Little & Vrijenhoek, 2003; Vrijenhoek, 2013) and there is potential confusion with morphologically similar polychaete tubes (Kiel & Dando, 2009).

An endemic tube-forming polychaete genus within extant hydrothermal vents on the East Pacific Rise (EPR) is *Alvinella* (Desbruyères & Laubier, 1986), comprising two species: *Alvinella pompejana* and *A. caudata*. Both are renowned for their occupation of high-temperature vent chimneys and role as biogeoengineers within this habitat (Desbruyères *et al*., 1998; Le Bris & Gaill, 2007). After colonization, *Alvinella* spp. can alter local vent fluid flow and composition, creating a range of micro-environments that allow the establishment of other hydrothermal vent biota less tolerant to high temperatures, and also promoting additional mineral precipitation and thus modifying chimney morphology (Juniper & Martineu, 1995; Pradillon *et al*., 2005). In part, this biological habitat modification arises from *Alvinella* spp. irrigating the interior of their tubes with cool sea water from above the alvinellid colony. This results in an inner tube environment with a lower temperature and a more neutral pH (temp. of ∼29–81 °C, pH ∼7) compared to conditions on the surface of the vent chimney substrate (temp. of ∼120 °C, pH ∼4) (Di Meo-Savoie *et al*., 2004; Le Bris *et al*., 2005) and creates buffered micro-niches which are colonized by micro-organisms (Le Bris *et al*., 2005).

Colonization of fresh vent chimneys by *Alvinella* spp. is considered to be strongly dependent on the properties of their unique tubes, which are attached directly onto vent chimney walls. These tubes possess high thermal and chemical stability (Gaill & Hunt, 1986) and can be secreted incredibly quickly, at a maximum rate of 1 cm day^−1^ in length (Pradillon *et al*., 2009). The tubes of *A. pompejana* and *A. caudata* are identical in appearance and are formed from granules primarily composed of protein (Vovelle & Gaill, 1986). The resulting tubes are fibrous and concentrically multilayered, with each tube layer comprised of superimposed sublayers of parallel fibrils that vary in direction between adjacent sublayers (Gaill & Hunt, 1986; Desbruyères *et al*., 1998). Both the inner and outer surfaces of *Alvinella* spp. tubes are covered by a patchy, but dense microbial community that includes filamentous, rod-shaped and coccoid forms (Desbruyères *et al*., 1985), belonging primarily to the epsilon subdivision of the proteobacteria (Haddad *et al*., 1995; Campbell & Cary, 2001; Campbell *et al*., 2003). Micro-organisms on the insides of the tubes can become trapped between the tube layers as more organic material is deposited during tube growth, to form distinctive microbial layers within the tube wall (Gaill & Hunt, 1986; Zbinden *et al*., 2001; Maginn *et al*., 2002).

Within the extreme environment of the EPR hydrothermal vents (see Fornari *et al*. (2012) and references therein for an overview of the EPR spreading centre), minerals can precipitate onto occupied *Alvinella* tubes remarkably quickly, such that an 11-day-old alvinellid colony can have 88% mineral content (Pradillon *et al*., 2009). During the early stages of this mineralization, minerals progressively coat the inner and outer tube surfaces (Gaill & Hunt, 1991) and accumulate within the tube walls, where they occur as nanocrystalline iron or zinc sulphides that assemble along sublayer surfaces (Zbinden *et al*., 2001, 2003; Maginn *et al*., 2002; Le Bris *et al*., 2008). Mineral precipitation has been observed particularly in tube layers containing trapped micro-organisms, and pyrite may occasionally replace organic tube layers (Maginn *et al*., 2002). Over time, *Alvinella* spp. tubes can become entirely mineral in composition (fully mineralized) (Haymon *et al*., 1984; Haymon & Koski, 1985). Full mineralization of originally organic polychaete tubes has also been observed by Cook & Stakes (1995) for siboglinid worm tubes at vent sites on the Juan de Fuca Ridge (JdFR), but the details of how *Alvinella* spp. tubes are fully mineralized, including the gross and fine-scale mineral textures and distributions, have not been documented.

Here, we provide a detailed account of the complete mineralization process of *Alvinella* spp. tubes to show how polychaete tubes can be fossilized at vent sites such as the EPR. A large number of *Alvinella* spp. tube specimens exhibiting varying degrees of mineralization have been analysed to better understand (i) the identity of the main minerals replacing the tubes, (ii) the nature and distribution of the mineral textures and (iii) the stages and timing of mineralization of the tubes. The identification of problematic tubular fossils from ancient vent sites is discussed, and mineralization of *Alvinella* spp. tubes is compared to preservation of organic tissues by silica and sulphide minerals within other environments.

## Methods

### Sample collection and storage

**Figure 1:**
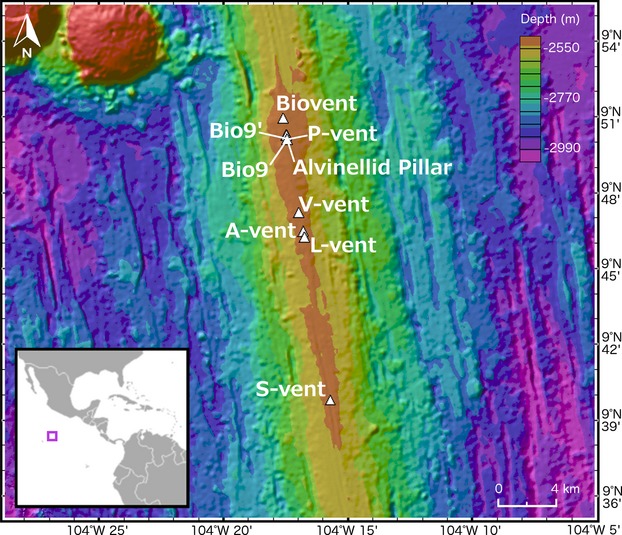
Location of the East Pacific Rise vent sites between 9° 41′ and 9° 51′ North from which the *Alvinella* spp. tube material was collected. *Insert*, Location of the study area in relation to Central America. The map was created using GeoMapApp©, and vent locations were plotted using data from the Marine Geoscience Data System (http://www.marine-geo.org/).

**Figure 2:**
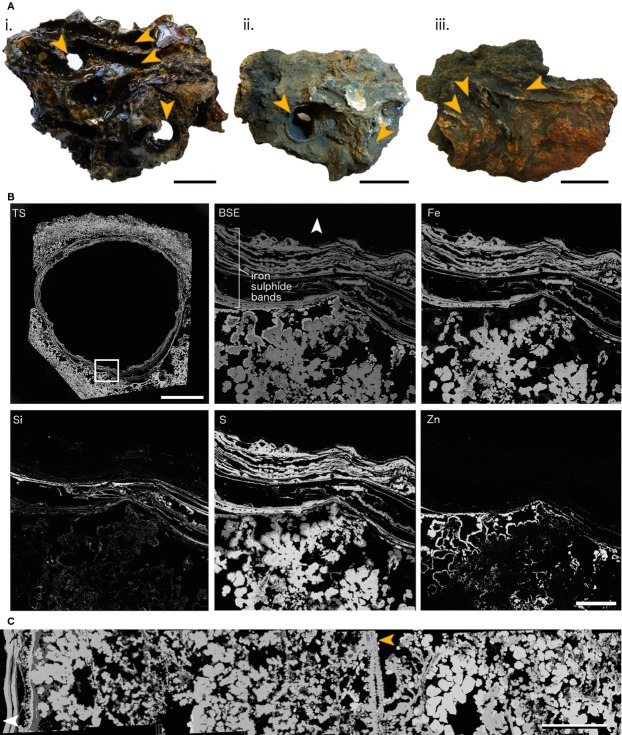
Fully mineralized *Alvinella* spp. tubes and associated vent chimney fragments. (A), Blocks of sulphide from vent chimneys containing fully mineralized *Alvinella* spp. tubes (orange arrows). (i, ii) Chimney blocks containing complex intertwined *Alvinella* spp. tubes, some of which have been mineralized completely as cylindrical structures; (iii) sulphide block with *Alvinella* spp. tubes mineralized on the surfaces that were attached to the vent chimney wall. (i) Specimen 74 (coated in epoxy resin); (ii) Specimen 57; (iii) Specimen 61. All scales in (A) are 30 mm. (B), WDS elemental mapping of a fully mineralized *Alvinella* spp. tube in transverse section (Polished Block 57.1) and associated vent chimney minerals. TS, Transverse section with the area analysed highlighted with white box; scale = 5 mm. BSE, Backscatter electron image of the analysed area; scale = 500 μm. Fe to Zn show the distribution of four elements within the area analysed (Fe – iron, Si – silicon, S – sulphur, Zn – zinc). (C), Backscatter SEM composite of Polished Block 60.2 showing variation in mineral texture and composition from a fully mineralized *Alvinella* spp. tube (extreme left) to several millimetres into the vent chimney. Brightest minerals = zinc sulphides, medium grey = iron sulphides and dullest grey = silica. The linear texture towards the middle of the image (orange arrow) likely represents an overgrown, older fully mineralized *Alvinella* spp. tube; scale = 1 mm. White arrows point towards tube centres.

The studied samples comprised vent chimney material containing *Alvinella* spp. tubes exhibiting varying degrees of mineralization. These were collected from the tops of nine active vent chimneys and one inactive chimney (Alvinellid Pillar) located along the EPR axial summit trough at depths of ∼2500 m (Fig.1). The material was collected on 10 dives of the submersible *Alvin*, during three Woods Hole Oceanographic Institution cruises of the RV Atlantis (AT15-13, AT15-27 and AT15-38, Table1). Some of the vent sites were sampled on more than one of the cruises (Table1), but different vent chimneys within these sites were sampled on each cruise. A small number of the studied samples were obtained through experimental fossilization cages, deployed at vent sites for approximately 1 year, during the same RV Atlantis cruises [outlined in Little (2009); see Methods S1]. After recovery from the sea floor, the *Alvinella* spp. tubes that were largely non-mineralized were removed from the vent chimneys and preserved in 95% ethanol (hereafter referred to as partially mineralized *Alvinella* spp. tubes). Samples of vent chimney sulphides with fully mineralized *Alvinella* spp. tubes were dried and stored at room temperature post-collection (Fig.2A). During post-collection storage, some of the sulphide chimney samples started to oxidize, forming secondary sulphate minerals; these were washed off prior to analyses. This oxidation may have resulted in the formation of iron oxides in addition to those formed *in situ* (*in situ* iron oxides were evidenced by a red colour on recovery; Fig.2A), and we hence excluded iron oxide analysis from the study.

**Table 1:** Information on the *Alvinella* spp. tube material used for this study. Specimen numbers were assigned during this study

Vent	Alvin dive	Collection date	Latitude of vent site	Longitude of vent site	Depth of vent site (m)	Temp. (°C)	pH	Specimens	Fossilization cage sample?
Bio9′	4274	24-Nov-06	N9° 50.311	W104° 17.480	2509	382	4.4	45, 66, 69	No
Bio9	4274	24-Nov-06	N9° 50.312	W104° 17.484	2509	388	3.6	55, 67, 71	No
	4375	11-Dec-07	N9° 50.312	W104° 17.484	2509	358	3.9	47, 56, 61, 70, 72	Yes – 370 days
L-vent	4276	26-Nov-06	N9° 46.256	W104° 16.749	2519	341	4.4	46, 54	No
	4377	13-Dec-07	N9° 46.256	W104° 16.749	2519	279	3.6	62, 65	No
	4467	01-Nov-08	N9° 46.256	W104° 16.749	2519	–	–	57, 60	Yes – 319 days
P-vent	4278	28-Nov-06	N9° 50.280	W104° 17.473	2509	392	4.5	44	No
Alvinellid Pillar	4281	01-Dec-06	N9° 50.125	W104° 17.456	2504	–	–	68	No
Biovent	4374	10-Dec-07	N9° 50.963	W104° 17.617	2501	349	4.1	49	No
A-vent	4377	13-Dec-07	N9° 46.500	W104° 16.810	2541	136	5.4	74	No
V-vent	4378	14-Dec-07	N9° 47.231	W104° 16.989	2517	363	3.6	48, 58	No
S-vent	4379	15-Dec-07	N9° 39.816	W104° 15.714	2510	326	4.3	59	No

Vent location, depth, temperature and pH data were obtained from the Marine Geoscience Data System (Bryce *et al*., 2007, 2008) (http://www.marine-geo.org/).

### Micro-CT analyses

Five partially mineralized *Alvinella* spp. tubes (Specimens 44, 46, 47, 48, 49; Table1) were initially scanned using a Metris X-Tek HMX ST 225 micro-computed tomography (μ-CT) system at the Natural History Museum, London, UK (NHM), to visualize the distribution of minerals on and within the tubes. Data volumes were constructed using CT Pro ver. 2.1 (Metris X-Tek, Tring, UK) and analysed using Drishti ver. 2.0 (Limaye, 2006). All five tube scans had a resolution of 71 μm or better. Mineral and organic tube components were separated based on grayscale values that represent X-ray attenuation, which closely corresponds to material density. To verify that the two were being accurately distinguished, one of the scanned specimens (Specimen 44) was embedded in resin, cross-sectioned and polished for reflected light microscopy to cross-reference the presence/absence of minerals with the CT scan reconstruction.

### Microscopic and chemical analyses

The ethanol-preserved tubes were critically point dried and, along with the fully mineralized tubes, were cut, impregnated in resin and made into polished blocks of both transverse and longitudinal tube sections. Polished blocks of mineralized *Alvinella* spp. tubes contained both tubes and a section of the surrounding vent chimney matrix. The polished blocks were coated with an approximately 17 nm carbon layer and imaged using the following scanning electron microscopes (SEM) with backscattered electron detectors: a LEO 1455VP SEM, a Carl Zeiss Ultra Plus Field Emission SEM, and an FEI Quanta 650 FEG-ESEM both at the NHM and at the University of Leeds, UK (Leeds). Two fully mineralized *Alvinella* spp. tubes (Specimens 54 and 55; Table1) were also imaged uncoated in the environmental chamber of a Philips XL 30 FEG-SEM at Leeds (UK).

The elemental composition of mineral phases, and elemental distribution were determined using both energy-dispersive X-ray spectroscopy (EDX) within the SEMs above, and wavelength-dispersive spectrometry (WDS) using a Cameca SX-100 electron microprobe (EPMA) at the NHM. An accelerating voltage of 20 kV was used for EDX point-analyses and maps, whereas in the EPMA, these were carried out using an accelerating voltage of 15 kV and a probe current of 40 nA for mapping and 20 nA for point analyses. Reflected light microscopy was used to identify the mineral phases within approximately half of the specimens (Table S1). X-ray diffraction (XRD) was performed on a Bruker D8 instrument (Bruker, Karlsruhe, Germany) (Cu Kα radiation source, 40 kV voltage and 40 mA of current) in Leeds on bulk material from a single vent chimney section and attached fully mineralized *Alvinella* spp. tube (Specimen 57) to identify the crystalline form of the zinc sulphide phase. In addition, confocal laser scanning microscopy (CLSM) using a Nikon A1-Si Confocal microscope at the NHM and operated in spectral imaging mode, was used to visualize the structure of the organic tube layers and microbial filaments on the inner surface of an *Alvinella* spp. tube (Specimen 44) by laser-induced autofluorescence.

### Measurements of mineral textures

The dimensions of mineral textures preserved within *Alvinella* spp. tubes were measured from SEM images using the program imagej (version 1.46r; National Institutes of Health, USA; http://rsb.info.nih.gov/ij). Pores and filaments were prevalent mineral textures within the samples, which are likely to be fossilized microbial filaments (see later). When measuring the dimensions of these textures, only pores with a distinctly circular or elliptical transverse section, that is those likely to be biogenic in origin, were measured. For statistical tests, diameter measurements from pore and filament textures were grouped into two types. Shapiro–Wilk normality tests were used to determine whether diameter measurements were normally distributed, and *F*-tests to compare variances between data pairs. Two-sample Kolmogorov–Smirnov tests were subsequently used to compare the cumulative distributions between pairs of diameter measurements. All three types of statistical test were performed in r (R Core Team, 2013).

## Results

### Vent chimney minerals around *Alvinella* spp. tubes

The fragments of vent chimneys onto which *Alvinella* spp. tubes were attached (Fig.2A) were formed largely of iron (pyrite, marcasite), zinc and copper (chalcopyrite, minor isocubanite) sulphides, silica, anhydrite and galena (Fig.2B,C). An XRD trace for a vent chimney sample with an attached tube (Specimen 57) showed the zinc sulphide to be sphalerite, but it is likely that both sphalerite and wurtzite were common in the samples (these polymorphs are difficult to discriminate when intergrown). The distribution of mineral phases within these vent chimney fragments was variable, but generally fine-grained marcasite occurred directly adjacent to *Alvinella* spp. tube walls on the outside of vent chimneys, which was sometimes overgrown by zinc sulphides (Fig.2B). This was succeeded by zinc sulphides and amorphous silica further into the vent chimney, which in turn was succeeded by larger-grained marcasite or zinc sulphides, then chalcopyrite or anhydrite (Fig.2C). The vent chimney minerals exhibited crystalline morphologies and porosity associated with fine-grained marcasite growth, while colloform (finely concentric and radiating) textures were rare and did not delineate consistent shapes. An exception were continuous thin bands of colloform iron sulphide (Fig.2C), found on the interiors of three chimney sections (Polished Blocks 57.3, 60.2 and 62.1). These were similar to the mineral layers comprising fully mineralized *Alvinella* spp. tubes (see later).

### Partially mineralized *Alvinella* spp. tubes

**Figure 3:**
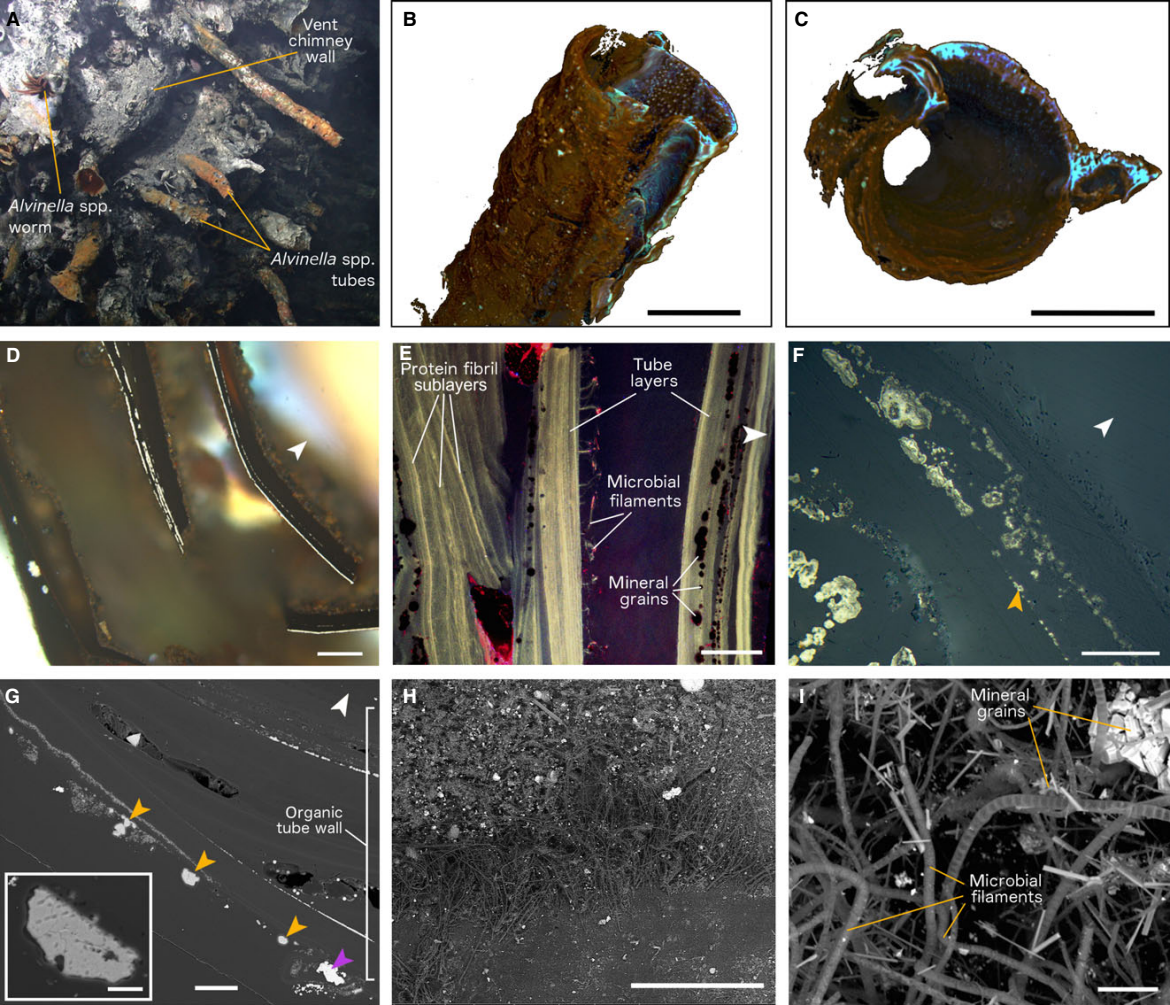
Partially mineralized *Alvinella* spp. tubes. (A) *Alvinella* spp. tubes on a hydrothermal vent chimney (L-vent, AT15-27, Alvin dive 4382), with an *Alvinella* spp. worm at its tube opening. Image credit: Woods Hole Oceanographic Institution. (B, C) Reconstructions of a single *Alvinella* spp. tube (Specimen 46) using micro-computed tomography (μ-CT). Blues and purples highlight dense areas where minerals have precipitated, while browns constitute the organic tube wall; scales = 10 mm. (B) tube in oblique side view; (C) tube in transverse section. (D) Bands of mineral growth within and on surfaces of organic *Alvinella* spp. tube layers, scale = 300 μm (Polished Block 46.1). (E) Confocal image of a transverse section through an *Alvinella* spp. tube (Polished Block 44.1), showing organic tube layers, microbial filaments trapped between layers, and the texture of protein fibrils within the organic tube. Scale = 100 μm. (F) Detail of an organic tube layer where mineralization begins as small iron sulphide cores, which join up upon further mineral precipitation to form distinct colloform pyrite bands. Cores and bands often occur along distinct surfaces within the organic layers (orange arrow) (Polished Block 44.1); scale = 50 μm. (G) Transverse section of an *Alvinella* spp. tube (Polished Block 44.1) with mineral grain (purple arrow) and elemental sulphur grains (orange arrows); scale = 100 μm. *Insert*, Detail of elemental sulphur grain showing pitted texture; scale = 20 μm. (H) SEM image of the interior surface of an *Alvinella* spp. tube (Specimen 44) showing patchily distributed microbial filaments and mineral grains, scale = 500 μm. (I) Detail of (H) scale = 10 μm. White arrows point towards tube centres.

Examples of *in situ* partially mineralized *Alvinella* spp. tubes are shown in Fig.3A. Three-dimensional μ-CT reconstructions of partially mineralized *Alvinella* spp. tubes showed that minerals were often concentrated along a longitudinal surface of the tubes (Fig.3B) (in Specimens 44, 46, 48, 49), which in one tube (Specimen 44) was known to have been the side that was directly attached to the vent chimney. Minerals occurred as grains and crusts coating inner and outer tube wall surfaces and were also abundant between the concentric organic layers that comprise the *Alvinella* spp. tube walls (Fig.3C). Detailed microscopy revealed that minerals were templating (here defined as the growth of minerals on a surface) certain organic tube layer and sublayer surfaces (Fig.3D–G), where mineral growth appears to begin as small cores, often <1 μm in diameter. These cores appeared to fuse with adjacent cores following further mineral precipitation, to form multiple bands of mineralization parallel to the natural layering of the tubes (Fig.3D–G). The composition of the minerals found within partially mineralized *Alvinella* spp. tubes generally reflected the mineralogy of vent chimney sections, with the mineral cores found to be predominantly comprised of iron sulphide, but occasionally also contained zinc and copper (Table S1). The cores tended to be larger and more abundant in the outer layers of tube walls and likely acted as loci for subsequent colloform iron sulphide growth. Large mineral grains, as well as large grains of elemental sulphur (Fig.3G), also occurred between organic tube layers and on both inner and outer tube surfaces. The elemental sulphur grains were usually 10 s of micrometres in size, but some were up to 468 μm across. They had a pitted texture (Fig.3G, insert) and were rarely observed in the fully mineralized tubes.

### Fully mineralized *Alvinella* spp. tubes

The fully mineralized *Alvinella* spp. tubes occurred in two forms: as tubes fully enclosed within vent chimney sulphides, in which the entire circumference of the tube wall had been preserved and tube interiors were mostly hollow (Fig.2A-i,ii), or as partial tube walls attached to the surfaces of vent chimney fragments (Fig.2A-iii). The fully mineralized *Alvinella* spp. tubes that were obtained from the fossilization experiment lasting approximately 1 year (319 and 370 days; Table1; Methods S1) demonstrate that full tube mineralization can occur within this time period.

**Figure 4:**
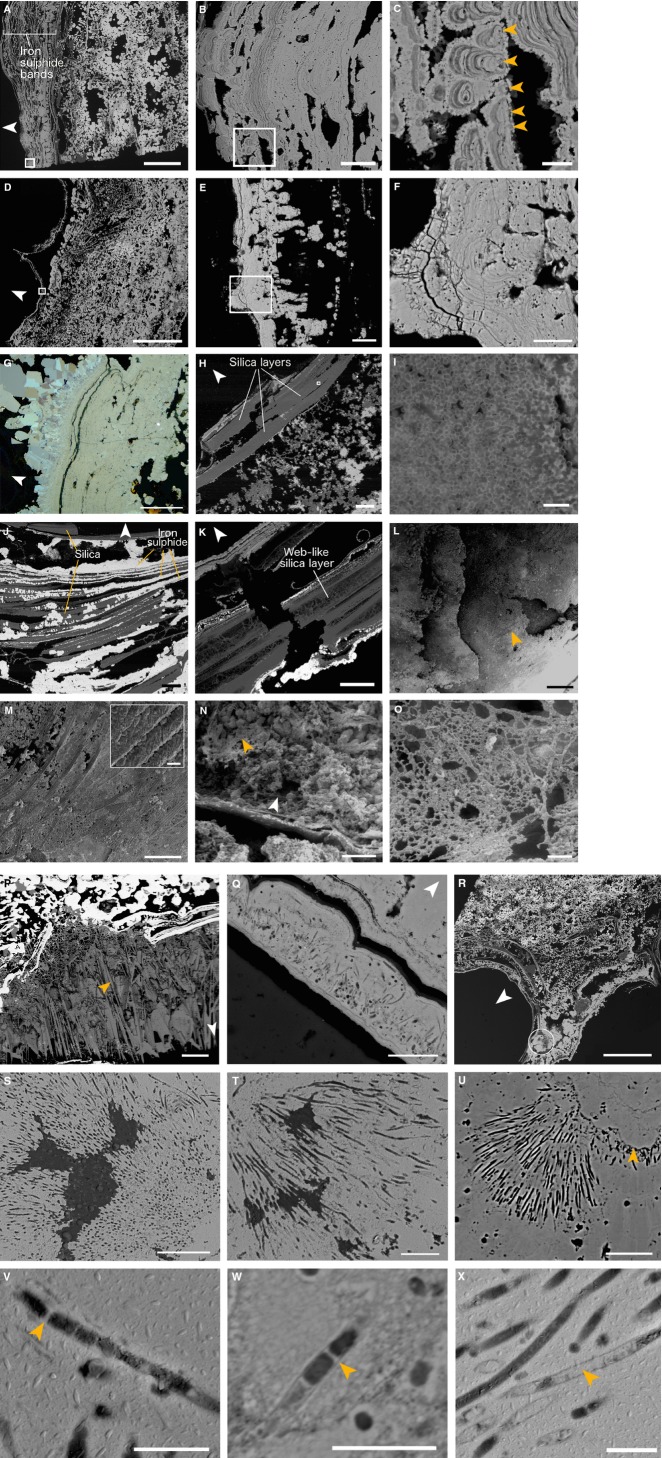
Fully mineralized *Alvinella* spp. tubes. (A) Longitudinal section of a tube (Polished Block 60.3) with a large number of iron sulphide (pyrite) bands replacing the tube wall; scale = 1 mm. White box shows location of (B). (B) Detail of boxed area in (A) showing bands of colloform pyrite; scale = 50 μm. White box shows location of (C). (C) Detail of boxed area in (B) showing colloform micro-stromatolitic structures with orange arrows pointing towards the cores from which they originate; scale = 10 μm. (D) Transverse section through two adjacent *Alvinella* spp. tubes; white box shows location of (E) scale = 4 mm. (E) Bands of pyrite comprising the mineralized tube, white box shows location of (F); scale = 50 μm. (F) Pore and filament textures within colloform pyrite (association 1); scale = 20 μm. (G) Bands of colloform pyrite overgrown by marcasite; scale = 100 μm (Polished Block 70.1). (H) Amorphous silica appears to be replacing organic tube layers (Polished Block 58.1). Scale = 200 μm; white box shows location of (I). (I) Detail of tube in (H) showing small silica spheres that comprise some of the amorphous silica layers; scale = 4 μm. (J) Interlaminated silica and pyrite, where silica appears to have preserved parts of disintegrating organic tube layers and surrounds iron sulphide cores (Polished Block 57.2). Scale = 200 μm. (K) Silica layer within a mineralized *Alvinella* spp. tube (Polished Block 62.1) exhibiting a web-like texture; scale = 200 μm. (L) View of the external wall of a fully mineralized *Alvinella* spp. tube (Specimen 54) showing four mineralized layers; orange arrow points to texture in (M). Scale = 500 μm. (M) detail of iron sulphide ‘fibres’ that are cross-cutting and/or bundled; scale = 250 μm. *Insert*, Detail of adjacent fibres showing surface covering of small cross-hatched striations; scale = 10 μm. (N) Interior of a mineralized *Alvinella* spp. tube (Specimen 55) which has been partially filled by minerals. The mineralized tube wall runs horizontally along the bottom third of the image; orange arrow points towards location of EPS-like mineral texture. Scale = 1 mm. (O) Detail of EPS-like mineral texture from the tube in (N); scale = 50 μm. (P), Anhydrite growing on the inside of an *Alvinella* spp. tube (orange arrow); scale = 500 μm. (Q), Pore and filament texture (association 1) occurring within one of the outer pyrite bands of a fully mineralized *Alvinella* spp. tube (Polished Block 57.1); scale = 20 μm. (R), Transverse section of *Alvinella* spp. tubes (Polished Block 60.1); white circle shows the location of clumped pores and filaments (association 2) in (S) and (T); scale = 500 μm. (S) Pore and filament clump showing a change in orientation from the clump base (bottom right) to the edge (left and top); scale = 20 μm. (T) Clumped radiating filaments; scale = 10 μm. (U) Clumped pore and filament association (Polished Block 63979) that appears to be rooted onto a distinct iron sulphide layer (orange arrow), scale = 20 μm. (V, W) Filaments from various samples with preserved septae (orange arrows). (V) Polished Block 60.1; (W) Polished Block 62.1. (X) Detail of filaments infilled by pyrite (orange arrow) (Polished Block 60.1). Scales in V–X are 3 μm. White arrows point towards tube centres.

The composition of fully mineralized *Alvinella* spp. tubes also reflected the mineralogy of adjacent vent chimney fragments. Mineral *Alvinella* spp. tube walls were mainly iron sulphide (pyrite and marcasite) and amorphous silica (Fig.2B; Table S1) in composition, with small quantities of zinc sulphides (sphalerite and/or wurtzite), and minor quantities of copper containing sulphides (chalcopyrite, isocubanite), galena and anhydrite. The majority of fully mineralized *Alvinella* spp. tubes were comprised of multilayered iron sulphide (pyrite) sheets that broadly mirrored the layering of organic tube walls, which appeared as concentric pyrite bands or horizons in transverse and longitudinal section (Figs2B,C and 4A–F). The pyrite bands occasionally showed weak anisotropy and contained overgrowths of crystalline marcasite that increased in crystal size away from the tube wall (Fig.4G).

The number of pyrite bands comprising fully mineralized *Alvinella* spp. tubes varied greatly between different tube samples (Table S1; Figs2B and 4A,D). Pyrite band number, thickness and the degree to which they joined with adjacent bands also varied between different parts of the same tube. The pyrite bands were characterized by colloform textures, the development of which could in some instances be traced to small iron sulphide cores very similar to those recorded within partially mineralized *Alvinella* spp. tubes (Figs3D–F and 4C,F). Sustained mineral precipitation onto the iron sulphide cores appears to have resulted in the formation of colloform micro-stromatolitic structures, up to 218 μm in length, comprised of fine-scale pyrite layers <1 μm thick (Fig.4B,C,E,F). Colloform layering tended to become increasingly sheet-like with distance away from the cores and as adjacent micro-stromatolitic structures fused (Fig.4B,E,G). Most micro-stromatolitic iron sulphide structures were oriented towards the inside of the tubes. Electron microprobe transects through the colloform textures (Tables S2–S5; Figs S2–S5) showed silicon or zinc to be present within some of the fine mineral layers; however, the small size of individual layers made it difficult to determine their elemental composition independently of the surrounding layers.

Amorphous silica was often present filling voids between pyrite bands in the fully mineralized *Alvinella* spp. tubes (Fig.2B). However, in four of the polished blocks (numbers 57.2, 57.3, 58, 62.1), the mineral tube wall was mainly comprised of layers of silica that resembled organic tube layers in thickness and shape (Fig.4H,I). These silica layers also contained rows of iron sulphide cores, much like those observed to have grown within the organic walls of partially mineralized *Alvinella* spp. tubes (Fig.4J). The silica layers were made up of small (<1 μm diameter) silica spheres, and in some places, the thick silica layers exhibited web-like or stringy textures (Fig.4J,K).

Additional mineral textures found within fully mineralized *Alvinella* spp. tubes comprised mainly of pyrite include a texture of cross-cutting and/or bundled ‘fibres’ (Fig.4L,M), which occurred on the external surface of a tube. Under higher magnification, these bundled ‘fibres’ showed a surface covering of smaller cross-hatched striations <1 μm in width (Fig.4M, insert). Another tube also contained mineral infilling of pyrite crystals, and a fine mesh-like structure also formed of pyrite (Fig.4N,O), while anhydrite was observed to have overgrown the inside of a different fully mineralized *Alvinella* spp. tube (Fig.4P).

### Pore and filament textures

**Figure 5:**
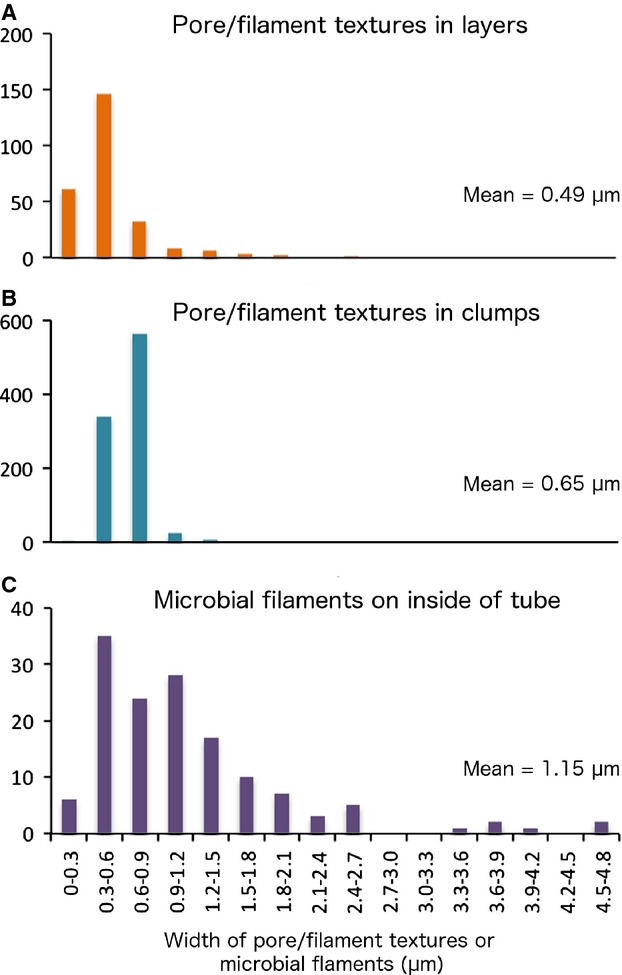
Frequency distribution plots showing diameter measurements for (A), pores and filaments occurring within mineralized bands of *Alvinella* spp. tubes (association 1); (B) those occurring as clumps (association 2); and (C) confirmed microbial filaments from the inside of an *Alvinella* spp. tube (Specimen 44).

Another texture prevalent in the fully mineralized tube samples was porosity. The pyrite minerals of several tubes contained circular pores 0.1 μm to several micrometres in diameter, in association with sinuous, unbranched filaments of a uniform diameter (Fig.4F,Q,S–U) (hereafter referred to as pore and filament associations). A few of these filaments contained cross-walls resembling septae (Fig.4V,W). Pore and filament textures were found to crosscut colloform structures, and in some samples, the filaments appeared ‘rooted’ to individual pyrite layers (Fig.4Q,U). Two types of pore and filament associations were identified, based on their mode of occurrence. The first (association 1) occurred within the pyrite layers comprising fully mineralized *Alvinella* spp. tubes (Fig.4F,Q) and had pores and filaments ranging from 0.13 to 2.62 μm in diameter (mean = 0.49 μm; Fig.5) that were in some instances very densely packed (Table S6). Association 1 filaments were hollow. The second type of pore and filament association (association 2) occurred as clumps of pores and filaments preserved within pyrite minerals adjacent to the outer layers of fully mineralized *Alvinella* spp. tubes (Fig.4R–U). Association 2 pores and filaments had diameters of a smaller size range (0.26–1.36 μm; mean = 0.65 μm) (Fig.5) and were often more densely packed than pores and filaments in association 1 (Table S6). Association 2 filaments at times also exhibited changes in orientation within the clumps, appearing in transverse section towards the centre of the clumps and in longitudinal section towards the clump perimeters (Fig.4S,U). The filaments were mostly hollow but some were infilled by pyrite (Fig.4X).

Confirmed microbial filaments occurring on the inner tube surface of a partially mineralized *Alvinella* spp. tube (Fig.3H,I) were measured for comparison with the two pore and filament associations and had a mean filament diameter of 1.15 μm (Fig.5). The three diameter measurement data sets (pores and filaments in layers; pores and filaments in clumps; and microbial filaments from an inner tube surface) were not normally distributed, and *F*-tests revealed the variances to be significantly different between all three data types (Table2). Subsequent two-sample Kolmogorov–Smirnov tests between the data type pairs were all significant (Table2). However, the *P*-values are approximate due to the presence of ties in the data.

**Table 2:** Results of statistical tests performed on pore and filament mineral textures preserved in mineral layers and as clumps, and microbial filaments from the inner surface of an *Alvinella* spp. tube (Specimen 44)

	P/F in layers	P/F in clumps	Microbial filaments
Shapiro–Wilk test			
*n*	259	939	142
*W*	0.7337	0.9550	0.8181
*P*	<2.2E-16	2.34E-16	5.47E-12

	P/F in clumps vs. P/F in layers	P/F in layers vs. microbial filaments	P/F in clumps vs. microbial filaments
*F*-test			
*F*	0.1861	0.1392	0.0259
*P*	0.000	0.000	0.000
Kolmogorov–Smirnov test			
*D*	0.5678	0.5584	0.5103
*P*	<2.2E-16*	<2.2E-16*	<2.2E-16*

* Due to the presence of ties, *P*-values are approximate. P/F = pores/filaments.

## Interpretations and Discussion

### *Alvinella* spp. tube mineralization process

#### Model of mineralization

**Figure 6:**
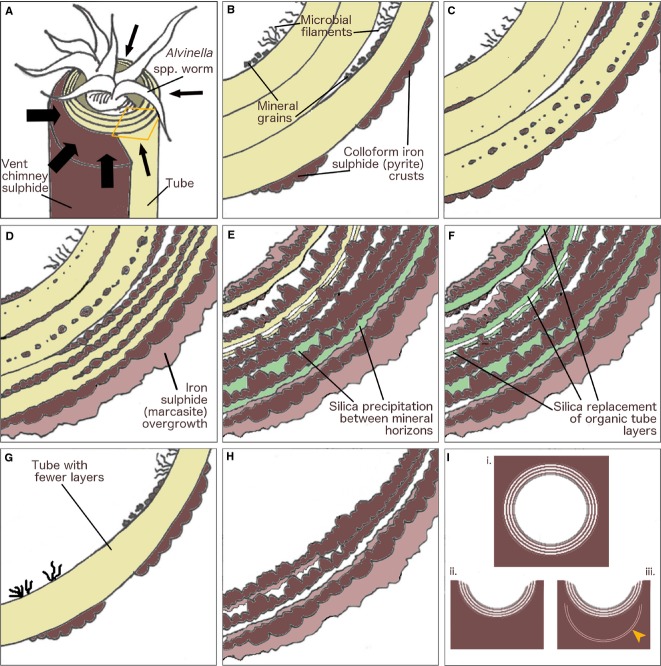
Stages of *Alvinella* spp. tube mineralization. (A) *Alvinella* spp. worm inside multilayered organic tube attached to vent chimney sulphide. Mineralization (black arrows) progresses from the outside of the tube towards the inside and is more prevalent from the vent chimney side. Orange area shows transverse section in (B–F). (B) Transverse section of tube in (A). Mineralization begins as colloform iron sulphide (pyrite) crusts forming on outer layers, and mineral grains becoming trapped in microbial filaments acting as nuclei for further mineral precipitation. (C), Minerals precipitate onto distinct surfaces within organic layers and sublayers, as small predominantly iron sulphide cores. (D), Further mineral precipitation results in the formation of concentric colloform pyrite bands. More crystalline iron sulphide (marcasite) overgrows initial iron sulphides deposited on the outside of the tube. (E), Organic matter degrades and silica occasionally fills gaps where it previously occurred. The supply of dissolved minerals changes to being from inside the tube, and growing colloform iron sulphides orient towards the tube interior. (F), In some instances, silica may directly preserve the degrading organic matter. Further overgrowth of more crystalline iron sulphide occurs. (G, H), Mineralization of a tube with fewer layers and without silica; (G) early stage; (H), late stage. (I) End products of *Alvinella* spp. tube mineralization recorded in this study. In transverse section: (i) full tube cylinder preserved; (ii) partial tube preservation; (iii) partial tube preservation with preservation of a previous tube beneath it (orange arrow).

Micro-CT reconstructions (Fig.3B,C) of the partially mineralized *Alvinella* spp. tubes show that tube mineralization begins preferentially along the longitudinal surfaces of the tubes which are attached to, or nearest to the vent chimney walls (Fig.6A). From these surfaces, mineralization likely spreads through the remainder of the tubes. This directional mineralization appears to result from a greater supply of mineral ions from the vent chimney. The greater amounts of sulphide minerals in the outer tube layers of partially mineralized tubes suggests that, at least initially, mineralization begins along the exterior surfaces of tubes. Mineralization appears to then progress within the organic walls of *Alvinella* spp. tubes, where fine iron sulphide (plus or minus a zinc and/or copper content) cores template tube layer and sublayer surfaces (Figs3D–F and 6C,D). Space to accommodate the growth of these cores may be provided by breaks between adjacent protein sublayers, possibly created by a poorer organization of their protein fibrils (Zbinden *et al*., 2001). The sulphide cores may also form within these particular layers because of an accumulation of metal ions (e.g. iron, Le Bris *et al*., 2008), and/or by seeding on mineral grains (including elemental sulphur) trapped between tube layers (Maginn *et al.,* 2002) (Figs3G and 6B,C). In addition, sulphide mineralization within *Alvinella* spp. tube walls may also be aided by the creation of oxygen-poor micro-environments between tube layers. Newly secreted *Alvinella* spp. tubes are permeable to hydrogen sulphide (Le Bris *et al*., 2008). If hydrogen sulphide is trapped within such crevices as it diffuses into the tube, a greater influence of anoxic vent fluid over sea water may favour the precipitation of sulphide minerals in tube wall interspaces.

The small sulphide mineral cores formed along the *Alvinella* spp. organic tube layer and sublayer surfaces continue to grow in a directed manner, amalgamating to produce bands of iron sulphides (pyrite). These run in parallel with the organic tube layering (Figs3C–F, 4A–F and 6D) and are often more numerous than the original organic tube layers. These pyrite bands show an increasingly colloform texture as they thicken, and can preserve fine-scale details of the original fibrous structure of the organic tubes (Fig.4L,M), possibly even individual protein fibres (Fig.4M, insert). The growing sulphide bands may also incorporate and fossilize the microbial community present between the organic tube layers, leading to the formation of layers of pyrite with pore and filament textures (as proposed by Maginn *et al*., 2002). Delamination of adjacent protein sublayers, probably resulting from iron sulphide growth and/or decay of the organic material, may expose additional surfaces for further templating by pyrite. This process could account for the very large number of pyrite bands observed in some of the fully mineralized tubes (Fig.4A,B). In other samples where the fully mineralized tubes comprise of only up to 3–5 mineral bands (Figs4D–F and 6G,H), the original organic tubes may not have been very thick, perhaps the result of a tube which was built and vacated fairly quickly by the worm [Zbinden *et al*. (2003) found 3–6 layers in 70-day-old *Alvinella* spp. tubes], or a tube that was being rapidly elongated to keep pace with rapid chimney growth as suggested by previous authors (Gaill & Hunt, 1991; Chevaldonne & Jollivet, 1993).

As the organic tube walls eventually decompose, sulphide mineral precipitation continues through the accretion of thin colloform pyrite layers onto existing mineral bands (Figs4B,C,E,F and 6E). At this stage, mineral precipitation appears to be greater on the inside of the tubes, which is shown by the orientation of micro-stromatolitic iron sulphide structures towards the tube interiors. The colloform pyrite bands that at this stage comprise the *Alvinella* spp. tube wall are subsequently overgrown by more crystalline marcasite (Figs4G and 6E,F,H). The mineral precipitation orientation change, and the growth of later marcasite minerals, can be explained by the absence of the worms at this stage of tube mineralization. During the life of *Alvinella* spp., the fluids in the tube are both cooler than end-member vent fluids and less acidic (pH ∼7 inside the tube compared to pH 4–5 outside; Le Bris *et al*., 2005), because of active mixing with sea water by the worms. However, once an *Alvinella* spp. worm has left its tube, the increasingly mineralized tubes can act as conduits for fluids that are closer in composition to end-member vent fluids, including being more acidic. Marcasite is precipitated preferentially to pyrite under conditions of lower pH (Murowchick & Barnes, 1986; Schoonen & Barnes, 1991a,b; Benning *et al*., 2000). Therefore, while a tube is occupied, the higher pH within the tube would result in preferential pyrite precipitation within the tube layers, which reflects mineralogical observations (Table S1). Some marcasite may, however, also be precipitated, while the worm is still occupying the tube. For example, in the case of the alvinellid *Paralvinella sulphincola*, marcasite is thought to be precipitated in local acidic conditions regulated by the production of tube mucus that is rich in elemental sulphur (Juniper & Sarrazin, 1995).

At this stage, silica may form layers between the pyrite bands (Fig.6E,F). This likely occurs due to convective cooling, which induces silica saturation and promotes its precipitation after the tube has already been mineralized by iron sulphides (Hannington & Scott, 1988; Juniper & Fouquet, 1988; Halbach *et al*., 2002). However, the silica layers that resemble organic tube layers and contain bands of iron sulphide cores (Fig.4H–K) suggest that the iron sulphide cores initially templated organic tube layers, which were later directly preserved by silica. These observations therefore suggest that silica can be intimately involved in the tube fossilization process, rather than occurring just as a late-stage mineral phase. These silica layers are unlikely to have formed through replacement (i.e. substitution of a mineral phase by another phase, or of organic matter by a mineral) of iron sulphides, as there is no evidence in our samples of the oxidized products (i.e. no iron oxides) that one might expect from this process.

The fully mineralized *Alvinella* spp. tubes may be preserved within the vent chimneys in several different forms (Fig.6I). Rapid mineral precipitation may surround entire tubes to create the porous structures in Fig.2A-i,ii, which are analogous to those observed by previous studies of EPR chimney sulphides (Hekinian *et al*., 1980; Haymon & Kastner, 1981; Haymon & Koski, 1985). Alternatively, only the sides of the tubes that are attached to the vent chimney may be mineralized, resulting in the partial tubes observed on mineral blocks used in this study (Fig.2A-iii). The concentric continuous layers of colloform pyrite that comprise fully mineralized *Alvinella* spp. tubes clearly distinguish them from vent chimney minerals, and such layers inside some of the vent chimney samples likely represent the mineralization of earlier tubes, which have been subsequently overgrown by vent chimney sulphides and other *Alvinella* spp. tubes (Fig.2C). Tubes may eventually be infilled by the precipitation of later-stage sulphides, including higher grade minerals (e.g. copper-rich sulphides), as noted by Cook & Stakes (1995) for mineralized siboglinid tubes from the JdFR.

The fully mineralized *Alvinella* spp. tubes collected from the fossilization experiment provide a maximum-time estimate for annelid tubes to be completely mineralized at hydrothermal vents (∼1 year). However, one should be cautious about deriving an actual rate of mineralization from the above, as it is not known at what point during the deployment period *Alvinella* spp. colonized the fossilization cages. Mineralization rates are also likely to be highly variable on small spatial scales due to the heterogeneous nature of vent environments (Van Dover, 2000). It is therefore very likely that *Alvinella* spp. tubes can become fully mineralized much more rapidly than within 1 year, as suggested by the very high mineral content observed within an 11-day-old alvinellid colony (Pradillon *et al*., 2009).

#### Microbial preservation

We interpret the pore and filament textures in pyrite found within and around the mineralized *Alvinella* spp. tubes to be the fossils of microbial filaments. This is because they demonstrate all of the suggested characteristics of *bona fide* microbial fossils (Westall, 1999; Schopf *et al*., 2010), are morphologically closely analogous to the microbial community associated with partially mineralized *Alvinella* spp. tubes (Gaill *et al*., 1987; Desbruyères *et al*., 1998; Zbinden *et al*., 2001; Maginn *et al*., 2002) and occur in a range of orientations throughout the matrix of the iron sulphides in which they are present (cf. pyrite leaching/oxidation by microbes; Verati *et al*., 1999; Edwards *et al*., 2003).

The microbial fossils appear to be mostly external filament moulds, but exhibit some evidence of replacement of septae and external sheaths by iron sulphide (Fig.4V–X), demonstrating that extremely fine-scale preservation can occur at hydrothermal vent sites. The difference in diameter distributions (Fig.5; Table2) between the fossil and the non-fossil microbial filaments may occur due to mineral replacement of microbial sheaths in the former. The significantly different diameter distributions between the fossil filaments occurring in iron sulphide bands (association 1) of the *Alvinella* spp. mineralized tube wall and those occurring as clumps around the tube (association 2) (Fig.5; Table2) are likely due to the fossilization of two distinct microbial communities. Micro-organisms sampled from whole *A. pompejana* tubes have been shown to differ to those sampled from tube interiors through molecular studies (Campbell *et al*., 2003), which likely reflects the variation in microhabitats between the tube interior and exterior. Microbial mats are commonly encountered on hydrothermal vent chimneys in areas colonized by *Alvinella* spp. (Taylor *et al*., 1999), and the position of the clumped filament fossils relative to the *Alvinella* spp. tubes in our samples indicates that the microbial filaments inhabited crevices next to the tubes, which may have provided some protection from thermal and chemical extremes. The marked differences in temperature and pH along an *Alvinella* spp. tube (temp. of ∼20 °C, pH ∼8 at tube openings; temp. of ∼120 °C, pH ∼4 adjacent to vent chimney substrate) (Le Bris *et al*., 2005) may explain the patchy distribution of the clumped filaments in our samples and why they were only found within a few of the examined specimens. The observation that the filaments in clumps are sometimes ‘rooted’ onto specific iron sulphide layers (Fig.4U) indicates a complex intergrowth of microbial mats and sulphide mineral precipitation.

The mesh-like pyritized structure associated with sulphide minerals precipitated in the internal space of the fully mineralized *Alvinella* spp. tube shown in Fig.4N,O likely represents mineralized EPS, due to the irregular sizes of the fibres comprising the mesh. In addition, the mesh in Fig.4O bears a strong resemblance to mineralized microbial EPS textures observed in hot spring deposits (Handley *et al*., 2008; Tobler *et al*., 2008; Peng & Jones, 2012). Many hydrothermal vent micro-organisms are known to secrete EPS (Raguenes *et al*., 1996, 1997b), including those associated with the integument of *A. pompejana* (Vincent *et al*., 1994; Raguenes *et al*., 1997a; Cambon-Bonavita *et al*., 2002). However, as the mesh observed in this study is positioned on top of minerals infilling an *Alvinella* spp. tube, we hypothesize that the mesh was created in the absence of an *Alvinella* spp. polychaete.

### Comparison with previous accounts of vent tube mineralization

Our observations of early-stage mineral precipitation in *Alvinella* spp. tubes are similar to that reported by Maginn *et al*. (2002), as we also observed early-stage iron sulphide mineralization along sublayers in *Alvinella* spp. tubes. However, we did not find pyrite to be directly replacing the organic walls of *Alvinella* spp. tubes in our samples, finding instead more evidence for the growth of sulphides on the organic layer surfaces (i.e. templating). The small iron sulphide cores we observed along tube sublayer surfaces appear analogous to the nanocrystalline zinc–iron sulphides reported by Zbinden *et al*. (2001, 2003). However, the general absence of zinc sulphides in our tube samples may be explained by different chemical and/or thermal characteristics of the vent fluids in the 9°N EPR area at the time that our samples were collected, compared to when the *Alvinella* spp. tubes studied by Zbinden *et al*. (2001, 2003) were obtained, as it is well established that EPR 9°N vent fluid temperature and chemistry vary temporally (Von Damm, 2000, 2004; Von Damm & Lilley, 2004). Previous studies on early-stage mineralization have also proposed that the presence of trapped micro-organisms within the organic tube walls of *Alvinella* spp. (Maginn *et al*., 2002) and siboglinid tubes (Peng *et al*., 2008, 2009) may have a direct control on their mineralization. However, mineral growth along the organic sublayers within our *Alvinella* spp. tubes often did not originate where trapped microbial filaments occurred, suggesting that mineralization may also take place in the absence of micro-organisms, in spaces between the protein fibril layering of *Alvinella* spp. tubes. This may also account for the absence of fossilized microbial filaments in many of the pyrite bands.

The fully mineralized *Alvinella* spp. tubes described here differ from the recently mineralized siboglinid worm tubes described by Cook & Stakes (1995) because at all stages of mineralization of the latter, the tubes are represented by a single layer of minerals and do not show the concentric multilayered banding of the *Alvinella* spp. tubes. This likely reflects the structural differences of siboglinid tubes compared to *Alvinella* spp. tubes (Gaill & Hunt, 1986; Shillito *et al*., 1995) and suggests that it may be possible to distinguish tubes from these two polychaete families in the fossil record, if their mineralized products are not substantially altered through diagenesis.

### Comparison with ancient hydrothermal vent tubeworm fossils

Our mineralized *Alvinella* spp. tubes differ in several respects from all fossil vent tubes described to date (Little *et al*., 1998). The Silurian vent tube species *Eoalvinellodes annulatus* (Little *et al*., 1999) occupied a similar ecological habitat as *Alvinella* spp., being found on the exterior of vent chimney walls, but the mineralized tubes of *E. annulatus* are formed of a single layer (0.04–0.60 mm thick) of framboidal and/or colloform pyrite, that is externally smooth but with internal ornament of concentric and subconcentric annulations. Further, *E. annulatus* tubes are generally smaller than *Alvinella* spp. tubes. The tubes of the Silurian vent species *Yamankasia rifeia* (Little *et al*., 1997, 1999) are equivalent in size to *Alvinella* spp. tubes and have tube walls formed of several thin layers (0.01–0.08 mm thick) of arsenian pyrite; however, they show an external ornament of concentric growth lines, fine longitudinal ridges that are absent in *Alvinella* spp. tubes. However, some *Y. rifeia* tube walls have a thick coating (up to 2.7 mm) of micro-laminated colloform pyrite with ∼1 μm diameter holes (Little *et al*., 1997), which are very much like the microbial fossils in *Alvinella* spp. tubes.

Haymon *et al*. (1984) and Haymon & Koski (1985) compared fully mineralized *Alvinella* spp. tubes from 21°N EPR with Upper Cretaceous (Bayda, Oman) tubular fossils formed of 2–3 concentric pyrite layers, with interlaminations of silica or void space. While these fossil tubes are morphologically similar to our *Alvinella* spp. tubes in terms of size and the presence of multilayering in the tube wall, they do not show all the characteristics outlined above for *Alvinella* spp. tubes, such as some tube walls being comprised of many more than three pyrite layers. In addition, the Bayda tubes exhibit annulations and longitudinal ornamentation which are more closely associated with siboglinid or chaetopterid tubes (Kiel & Dando, 2009; Hilário *et al*., 2011) and are generally absent from *Alvinella* spp. tubes. Because of this, we suggest that no known ancient vent fossil tubes are equivalent to present day *Alvinella* spp. tubes. This interpretation is currently supported by molecular evidence suggesting that modern alvinellids diverged from 41 to 51 Ma (Eocene) (Vrijenhoek, 2013), whereas the majority of well-preserved tubular vent fossils are Mesozoic or Palaeozoic in age (Little *et al*., 1997, 1998, 1999).

### Soft tissue preservation by silica and pyrite

Our study shows that the proteinaceous organic tube walls of *Alvinella* spp. and associated microbial cells can be rapidly preserved by both sulphide minerals and silica at vent sites. Soft tissue preservation by pyrite is on the whole rare and only seen at sites of exceptional preservation, for example Beecher's Trilobite Bed, Upper Ordovician, New York State, USA, or the Hunsrückschiefer, Devonian, western Germany (Briggs *et al*., 1991; Briggs & Bartels, 2001; Raiswell *et al*., 2008; Farrell *et al*., 2013). In these cases, soft tissues are preserved by an infilling or templating of pyrite occurring as framboids, pyritohedra and euhedral crystals generally <20 μm in size (Briggs *et al*., 1991, 1996). Sulphide mineralization within this setting is biogenically mediated as it results from the decay of the soft tissues and leads to the localization of sulphide mineral precipitation on and around these tissues. Within hydrothermal vent environments, mineral precipitation generally occurs abiogenically as a result of the mixing of hot, mineral-rich vent fluid with cooler sea water. However, the role of decay products on mineralization within vents is largely unknown. Elevated dissolved sulphide, low oxygen and low pH conditions that may be especially concentrated around decomposing *Alvinella* spp. tube surfaces could act to induce local supersaturation of iron and sulphide at these sites and thereby promote more intense iron sulphide precipitation. The micro-stromatolitic, fine-scale nature of pyrite that preserves *Alvinella* spp. tubes could be indicative of this process, as small pyrite grain sizes suggest abundant nucleation, which is considered a product of high degrees of supersaturation and the formation of colloform pyrite (Barrie *et al*., 2009). Decay-induced local supersaturation may thereby account for the widespread association and organization of colloform iron sulphide textures with *Alvinella* spp. tubes, compared to their relative rarity within vent chimney minerals. The role of decay products in hydrothermal vent mineralization therefore could have similarities to (albeit different products to) pyritization within soft sediments, but warrants further investigation.

Within terrestrial hydrothermal systems (hot springs), detailed preservation of organic matter by early-stage silica microspheres has been widely documented for micro-organisms (Westall *et al*., 1995; Jones *et al*., 1997, 1998; Konhauser *et al*., 2001) as well as for wood (Akahane *et al*., 2004). The mechanisms of preservation can be diverse, and both micro-organisms and plant tissues may be replaced or templated by silica (Jones & Renaut, 2003), or silica can infill the lumina of plant cells (Akahane *et al*., 2004). Analogous mechanisms might be proposed for silica mineralization within the similar setting of deep-sea hydrothermal vent environments. In the *Alvinella* spp. tubes, the mechanism through which silica appears to have preserved organic tube walls does not seem to be void filling, as silica mimics the appearance of tube layers rather than gaps between or within them. Instead, silica seems to have either replaced organic matter directly, or to have universally templated protein fibres within the tube walls. These modes of mineralization can account for the stringy, web-like appearance of silica in some of the fully mineralized tubes (Fig.4K). As in terrestrial hot springs, several modes of organic matter preservation by silica may be occurring simultaneously within hydrothermal vents, but if silica templating is a pathway through which *Alvinella* spp. tubes are mineralizing, it seems to be occurring in an altogether different manner to pyrite templating.

## Conclusions

Documenting in detail the mineralization of modern polychaete tubes is critical to ensuring the validity of fossil-modern comparisons and to advancing current understanding of the taphonomy and palaeontology of polychaete worms. We have shown how *Alvinella* tubes can be fully mineralized within a modern hydrothermal vent setting as multiple concentric pyrite bands that include fine-scale features such as protein fibres and associated micro-organisms. Our ability to interpret ancient fossils will always be limited by paucity of material and diagenetic alteration, and it is important to be mindful of these factors when comparing ancient to recently mineralized material. Fortunately, many ancient hydrothermal vent tube fossils appear well preserved, with some of the oldest specimens exhibiting fine colloform layering which suggests that they have not been significantly altered. These tubes will make ideal targets for investigating the preservation and nature of interactions between micro-organisms and tubeworms, a largely unknown aspect of the ecology of ancient vent communities.

## Supporting Information

Fig S1 The vent fossilization experiment from which some of the samples used in this study were obtained.

Fig S2 Locations of EPMA point analyses for colloform mineral textures (polished Block 57.1).

Fig S3 Locations of EPMA point analyses for colloform mineral textures (polished Block 57.1).

Fig S4 Locations of EPMA point analyses for colloform mineral textures (polished Block 57.1).

Fig S5 Locations of EPMA point analyses for colloform mineral textures (polished Block 57.1).

**Table S1.**Mineralogical observations for each of the polished blocks used in this study. **Table S2.**EPMA point analyses for colloform mineral textures (polished Block 57.1). **Table S3.**EPMA point analyses for colloform mineral textures (polished Block 57.1). **Table S4.**EPMA point analyses for colloform mineral textures (polished Block 57.1). **Table S5.**EPMA point analyses for colloform mineral textures (polished Block 57.1). **Table S6.**Information on the diameter measurements of pore and filament associations found within fully mineralized *Alvinella* spp. tubes, and of microbial filaments from the inner surface of an *Alvinella* spp. tube (Specimen 44). **Methods S1.**Fossilization cage experiment.

## References

[b1] Akahane H, Furuno T, Miyajima H, Yoshikawa T, Yamamoto S (2004). Rapid wood silicification in hot spring water: an explanation of silicification of wood during the Earth's history. Sedimentary Geology.

[b2] Barrie CD, Boyce AJ, Boyle AP, Williams PJ, Blake K, Ogawara T, Akai J, Prior DJ (2009). Growth controls in colloform pyrite. American Mineralogist.

[b3] Benning L, Wilkin R, Barnes H (2000). Reaction pathways in the Fe–S system below 100°C. Chemical Geology.

[b4] Boyce AJ, Little CTS, Russell MJ (2003). A new fossil vent biota in the Ballynoe barite deposit, Silvermines, Ireland: evidence for intracratonic sea-floor hydrothermal activity about 352 Ma. Economic Geology.

[b5] Briggs DEG, Bartels C (2001). New arthropods from the Lower Devonian Hunsrück Slate (Lower Emsian, Rhenish Massif, Western Germany). Palaeontology.

[b6] Briggs DEG, Bottrell SH, Raiswell R (1991). Pyritization of soft-bodied fossils: Beecher's Trilobite Bed, Upper Ordovician, New York State. Geology.

[b7] Briggs DEG, Raiswell R, Bottrell SH, Hatfield DT, Bartels C (1996). Controls on the pyritization of exceptionally preserved fossils; an analysis of the Lower Devonian Hunsrück Slate of Germany. American Journal of Science.

[b8] Bryce J, Prado F, Von Damm K (2007). http://www.marine-geo.org/tools/search/Files.php?data_set_uid=17364.

[b9] Bryce J, Prado F, Von Damm K (2008). http://www.marine-geo.org/tools/search/Files.php?data_set_uid=17603.

[b10] Cambon-Bonavita M-A, Raguenes G, Jean J, Vincent P, Guezennec J (2002). A novel polymer produced by a bacterium isolated from a deep-sea hydrothermal vent polychaete annelid. Journal of Applied Microbiology.

[b11] Campbell BJ, Cary SC (2001). Characterization of a novel spirochete associated with the hydrothermal vent polychaete annelid, *Alvinella pompejana*. Applied and Environmental Microbiology.

[b12] Campbell B, Stein J, Cary S (2003). Evidence of chemolithoautotrophy in the bacterial community associated with *Alvinella pompejana*, a hydrothermal vent polychaete. Applied and Environmental Microbiology.

[b13] Chevaldonne P, Jollivet D (1993). Videoscopic study of deep-sea hydrothermal vent alvinellid polychaete populations: biomass estimation and behaviour. Marine Ecology Progress Series.

[b14] Conway Morris S, Peel JS (2008). The earliest annelids: lower Cambrian polychaetes from the Sirius Passet Lagerstätte, Peary Land, North Greenland. Acta Palaeontologica Polonica.

[b15] Cook T, Stakes D (1995). Biogeological mineralization in deep-sea hydrothermal deposits. Science.

[b16] Desbruyères D, Gaill F, Laubier L, Fouquet Y, Jones ML (1985). Polychaetous annelids from hydrothermal vent ecosystems: an ecological overview. Hydrothermal Vents of the Eastern Pacific: An Overview.

[b17] Desbruyères D, Chevaldonné P, Alayse A-M, Jollivet D, Lallier FH, Jouin-Toulmond C, Zal F, Sarradin P-M, Cosson R, Caprais J-C, Arndt C, O'Brien J, Guezennec J, Hourdez S, Riso R, Gaill F, Laubier L, Toulmond A (1998). Biology and ecology of the ‘Pompeii worm’ (*Alvinella pompejana* Desbruyères and Laubier), a normal dweller of an extreme deep-sea environment: a synthesis of current knowledge and recent developments. Deep Sea Research II.

[b18] Di Meo-Savoie CA, Luther GW, Cary SC (2004). Physicochemical characterization of the microhabitat of the epibionts associated with *Alvinella pompejana*, a hydrothermal vent annelid. Geochimica et Cosmochimica Acta.

[b19] Edwards KJ, McCollom T, Konishi H, Buseck P (2003). Seafloor bioalteration of sulfide minerals: results from in situ incubation studies. Geochimica et Cosmochimica Acta.

[b20] Farrell ÚC, Briggs DEG, Hammarlund EU, Sperling EA, Gaines RR (2013). Paleoredox and pyritization of soft-bodied fossils in the Ordovician Frankfort Shale of New York. American Journal of Science.

[b21] Fornari DJ, Von Damm KL, Bryce JG, Cowen JP, Ferrini V, Fundis A, Lilley MD, Luther GW, Mullineaux LS, Perfit MR, Meana-Prado MF, Rubin KH, SeyfriedJr WE, Shank TM, Soule SA, Tolstoy M, White SM (2012). The East Pacific Rise between 9°N and 10°N: twenty-five years of integrated, multidisciplinary oceanic spreading center studies. Oceanography.

[b22] Gaill F, Hunt S (1986). Tubes of deep sea hydrothermal vent worms *Riftia pachyptila* (Vestimentifera) and *Alvinella pompejana* (Annelida). Marine Ecology Progress Series.

[b23] Gaill F, Hunt S (1991). The biology of annelid worms from high temperature hydrothermal vent regions. Reviews in Aquatic Sciences.

[b24] Gaill F, Desbruyères D, Prieur D (1987). Bacterial communities associated with “Pompei Worms” from the East Pacific Rise hydrothermal vents: SEM, TEM observations. Microbial Ecology.

[b25] Haddad A, Camacho F, Durand P, Cary SC (1995). Phylogenetic characterization of the epibiotic bacteria associated with the hydrothermal vent polychaete *Alvinella pompejana*. Applied and Environmental Microbiology.

[b26] Halbach M, Halbach P, Luders V (2002). Sulfide-impregnated and pure silica precipitates of hydrothermal origin from the Central Indian Ocean. Chemical Geology.

[b27] Handley KM, Turner SJ, Campbell KA, Mountain BW (2008). Silicifying biofilm exopolymers on a hot-spring microstromatolite: templating nanometer-thick laminae. Astrobiology.

[b28] Hannington MD, Scott SD (1988). Mineralogy and geochemistry of a hydrothermal silica-sulfide-sulfate in the caldera of Axial Seamount, Juan de Fuca ridge. Canadian Mineralogist.

[b29] Haymon RM, Kastner M (1981). Hot spring deposits on the East Pacific Rise at 21°N: preliminary description of mineralogy and genesis. Earth and Planetary Science Letters.

[b30] Haymon R, Koski R (1985). Evidence of an ancient hydrothermal vent community: fossil worm tubes in Cretaceous sulfide deposits of the Samail Ophiolite, Oman. Bulletin of the Biological Society of Washington.

[b31] Haymon RM, Koski RA, Sinclair C (1984). Fossils of hydrothermal vent worms from Cretaceous sulfide ores of the Samail Ophiolite, Oman. Science.

[b32] Hekinian R, Fevrier M, Bischoff JL, Picot P, Shanks W (1980). Sulfide deposits from the East Pacific Rise near 21°N. Science.

[b33] Hilário A, Capa M, Dahlgren TG, Halanych KM, Little CTS, Thornhill DJ, Verna C, Glover AG (2011). New perspectives on the ecology and evolution of siboglinid tubeworms. PLoS ONE.

[b34] Jones B, Renaut RW (2003). Hot spring and geyser sinters: the integrated product of precipitation, replacement, and deposition. Canadian Journal of Earth Sciences.

[b35] Jones B, Renaut RW, Rosen MR (1997). Biogenicity of silica precipitation around geysers and hot-spring vents, North Island, New Zealand. Journal of Sedimentary Research.

[b36] Jones B, Renaut RW, Rosen MR (1998). Microbial biofacies in hot-spring sinters: a model based on Ohaaki Pool, North Island, New Zealand. Journal of Sedimentary Research.

[b37] Juniper SK, Fouquet Y (1988). Filamentous iron-silica deposits from modern and ancient hydrothermal sites. Canadian Mineralogist.

[b38] Juniper S, Martineu P (1995). Alvinellids and sulfides at hydrothermal vents of the eastern Pacific: a review. American Zoologist.

[b39] Juniper SK, Sarrazin J, Humphris SE, Zierenberg RA, Mullineaux LS, Thomson RE (1995). Interaction of vent biota and hydrothermal deposits: present evidence and future experimentation. Seafloor Hydrothermal Systems: Physical, Chemical, Biological, and Geological Interactions.

[b40] Kiel S, Dando PR (2009). Chaetopterid tubes from vent and seep sites: implications for fossil record and evolutionary history of vent and seep annelids. Acta Palaeontologica Polonica.

[b41] Konhauser KO, Phoenix RR, Bottrell SH, Adams DG, Head IM (2001). Microbial–silica interactions in Icelandic hot spring sinter: possible analogues for some Precambrian siliceous stromatolites. Sedimentology.

[b42] Le Bris N, Gaill F (2007). How does the annelid *Alvinella pompejana* deal with an extreme hydrothermal environment?. Reviews in Environmental Science and Biotechnology.

[b43] Le Bris N, Zbinden M, Gaill F (2005). Processes controlling the physico-chemical micro-environments associated with Pompeii worms. Deep Sea Research I.

[b44] Le Bris N, Anderson L, Chever F, Gaill F, Mertens LP (2008). Sulfide diffusion and chemoautotrophy requirements in an extremophilic worm tube. Biological Oceanography Research Trends.

[b45] Limaye A (2006). Drishti: Volume Exploration and Presentation Tool.

[b46] Little CTS (2009). Planet Earth Online.

[b47] Little CTS, Vrijenhoek RC (2003). Are hydrothermal vent animals living fossils?. Trends in Ecology & Evolution.

[b48] Little CTS, Herrington RJ, Maslennikov V, Morris NJ, Zaykov V (1997). Silurian hydrothermal-vent community from the southern Urals, Russia. Nature.

[b49] Little CTS, Herrington RJ, Maslennikov V, Zaykov V (1998). The fossil record of hydrothermal vent communities. Geological Society, London, Special Publications.

[b50] Little CTS, Maslennikov V, Morris NJ, Gubanov AP (1999). Two Palaeozoic hydrothermal vent communities from the southern Ural mountains, Russia. Palaeontology.

[b51] Little CTS, Danelian T, Herrington RJ, Haymon R (2004). Early Jurassic hydrothermal vent community from the Franciscan Complex, California. Journal of Paleontology.

[b52] Little CTS, Magalashvili A, Banks D (2007). Neotethyan Late Cretaceous volcanic arc hydrothermal vent fauna. Geology.

[b53] Maginn E, Little CTS, Herrington R, Mills R (2002). Sulphide mineralisation in the deep sea hydrothermal vent polychaete, *Alvinella pompejana*: implications for fossil preservation. Marine Geology.

[b54] Murowchick J, Barnes H (1986). Marcasite precipitation from hydrothermal solutions. Geochimica et Cosmochimica Acta.

[b55] Peng X, Jones B (2012). Rapid precipitation of silica (opal-A) disguises evidence of biogenicity in high-temperature geothermal deposits: case study from Dagunguo hot spring, China. Sedimentary Geology.

[b56] Peng X, Zhou H, Tang S, Yao H, Jiang L, Wu Z (2008). Early-stage mineralization of hydrothermal tubeworms: new insights into the role of microorganisms in the process of mineralization. Chinese Science Bulletin.

[b57] Peng X, Zhou H, Yao H, Li J, Wu Z (2009). Ultrastructural evidence for iron accumulation within the tube of Vestimentiferan *Ridgeia piscesae*. BioMetals.

[b58] Pradillon F, Zbinden M, Mullineaux L, Gaill F (2005). Colonisation of newly-opened habitat by a pioneer species, *Alvinella pompejana* (Polychaeta: Alvinellidae), at East Pacific Rise vent sites. Marine Ecology Progress Series.

[b59] Pradillon F, Zbinden M, Le Bris N, Hourdez S, Barnay A-S, Gaill F (2009). Development of assemblages associated with alvinellid colonies on the walls of high-temperature vents at the East Pacific Rise. Deep Sea Research II.

[b60] R Core Team (2013). R: A Language and Environment for Statistical Computing.

[b61] Raff EC, Schollaert KL, Nelson DE, Donoghue PC, Thomas CW, Turner FR, Stein BD, Dong X, Bengtson S, Huldtgren T, Stampanoni M, Chongyu Y, Raff RA (2008). Embryo fossilization is a biological process mediated by microbial biofilms. Proceedings of the National Academy of Sciences.

[b62] Raguenes G, Pignet P, Gauthier G, Peres A, Christen R, Rougeaux H, Barbier G, Guezennec J (1996). Description of a new polymer-secreting bacterium from a deep-sea hydrothermal vent, *Alteromonas macleodii* subsp. *fijiensis*, and preliminary characterization of the polymer. Applied and Environmental Microbiology.

[b63] Raguenes G, Christen R, Guezennec JG, Pignet P, Barbier G (1997a). *Vibrio diabolicus* sp. nov., a new polysaccharide-secreting organism isolated from a deep-sea hydrothermal vent polychaete annelid, *Alvinella pompejana*. International Journal of Systematic Bacteriology.

[b64] Raguenes G, Peres A, Ruimy R, Pignet P, Christen R, Loaec M, Rougeaux H, Barbier G, Guezennec J (1997b). Alteromonas infernus sp. nov., a new polysaccharide-producing bacterium isolated from a deep-sea hydrothermal vent. Journal of Applied Microbiology.

[b65] Raiswell R, Newton R, Bottrell SH, Coburn PM, Briggs DEG, Bond DP, Poulton SW (2008). Turbidite depositional influences on the diagenesis of Beecher's Trilobite Bed and the Hunsrück Slate; sites of soft tissue pyritization. American Journal of Science.

[b66] Schoonen M, Barnes H (1991a). Reactions forming pyrite and marcasite from solution: I. Nucleation of FeS2 below 100°C. Geochimica et Cosmochimica Acta.

[b67] Schoonen M, Barnes H (1991b). Reactions forming pyrite and marcasite from solution: II. Via FeS precursors below 100°C. Geochimica et Cosmochimica Acta.

[b68] Schopf JW, Kudryavtsev AB, Sugitani K, Walter MR (2010). Precambrian microbe-like pseudofossils: a promising solution to the problem. Precambrian Research.

[b69] Shillito B, Lechaire J-P, Goffinet G, Gaill F (1995). Composition and morphogenesis of the tubes of vestimentiferan worms. Geological Society, London, Special Publications.

[b70] Taylor CD, Wirsen CO, Gaill F (1999). Rapid microbial production of filamentous sulfur mats at hydrothermal vents. Applied and Environmental Microbiology.

[b71] Tobler DJ, Stefánsson A, Benning LG (2008). In-situ grown silica sinters in Icelandic geothermal areas. Geobiology.

[b72] Van Dover C (2000). The Ecology of Deep-Sea Hydrothermal Vents.

[b73] Verati C, De Donato P, Prieur D, Lancelot J (1999). Evidence of bacterial activity from micrometer-scale layer analyses of black-smoker sulfide structures (Pito Seamount Site, Easter microplate). Chemical Geology.

[b74] Vincent P, Pignet P, Talmont F, Bozzi L, Fournet B, Guezennec J, Jeanthon C, Prieur D (1994). Production and characterization of an exopolysaccharide excreted by a deep-sea hydrothermal vent bacterium isolated from the polychaete annelid *Alvinella pompejana*. Applied and Environmental Microbiology.

[b75] Vinther J, Eibye-Jacobsen D, Harper DA (2011). An Early Cambrian stem polychaete with pygidial cirri. Biology Letters.

[b76] Von Damm KL (2000). Chemistry of hydrothermal vent fluids from 9°-10°N, East Pacific Rise: “Time zero,” the immediate posteruptive period. Journal of Geophysical Research.

[b77] Von Damm KL (2004). Evolution of the hydrothermal system at East Pacific Rise 9°50′ N: geochemical evidence for changes in the upper oceanic crust. Geophysical Monograph Series.

[b78] Von Damm KL, Lilley MD (2004). Diffuse flow hydrothermal fluids from 9°50′ N East Pacific Rise: origin, evolution and biogeochemical controls. Geophysical Monograph Series.

[b79] Vovelle J, Gaill F (1986). Données morphologiques, histochimiques et microanalytiques sur l'élaboration du tube organo-minéral d'*Alvinella pompejana*, polychète des sources hydrothermales, et leurs implications phylogénétiques. Zoologica Scripta.

[b80] Vrijenhoek RC (2013). On the instability and evolutionary age of deep-sea chemosynthetic communities. Deep Sea Research II.

[b81] Westall F (1999). The nature of fossil bacteria: a guide to the search for extraterrestrial life. Journal of Geophysical Research.

[b82] Westall F, Boni L, Guerzoni E (1995). The experimental silicification of microorganisms. Palaeontology.

[b83] Zbinden M, Martinez I, Guyot F, Cambon-Bonavita M-A, Gaill F (2001). Zinc-iron sulphide mineralization in tubes of hydrothermal vent worms. European Journal of Mineralogy.

[b84] Zbinden M, Le Bris N, Compère P, Martinez I, Guyot F, Gaill F (2003). Mineralogical gradients associated with alvinellids at deep-sea hydrothermal vents. Deep Sea Research Part I: Oceanographic Research Papers.

