# Supplementary figures and images for: Mineralization of Alvinella polychaete tubes at hydrothermal vents
Source: Geobiology. 2014 Dec 30;13(2):152–69. doi: 10.1111/gbi.12123 (PMC4359681; doi:10.1111/gbi.12123)

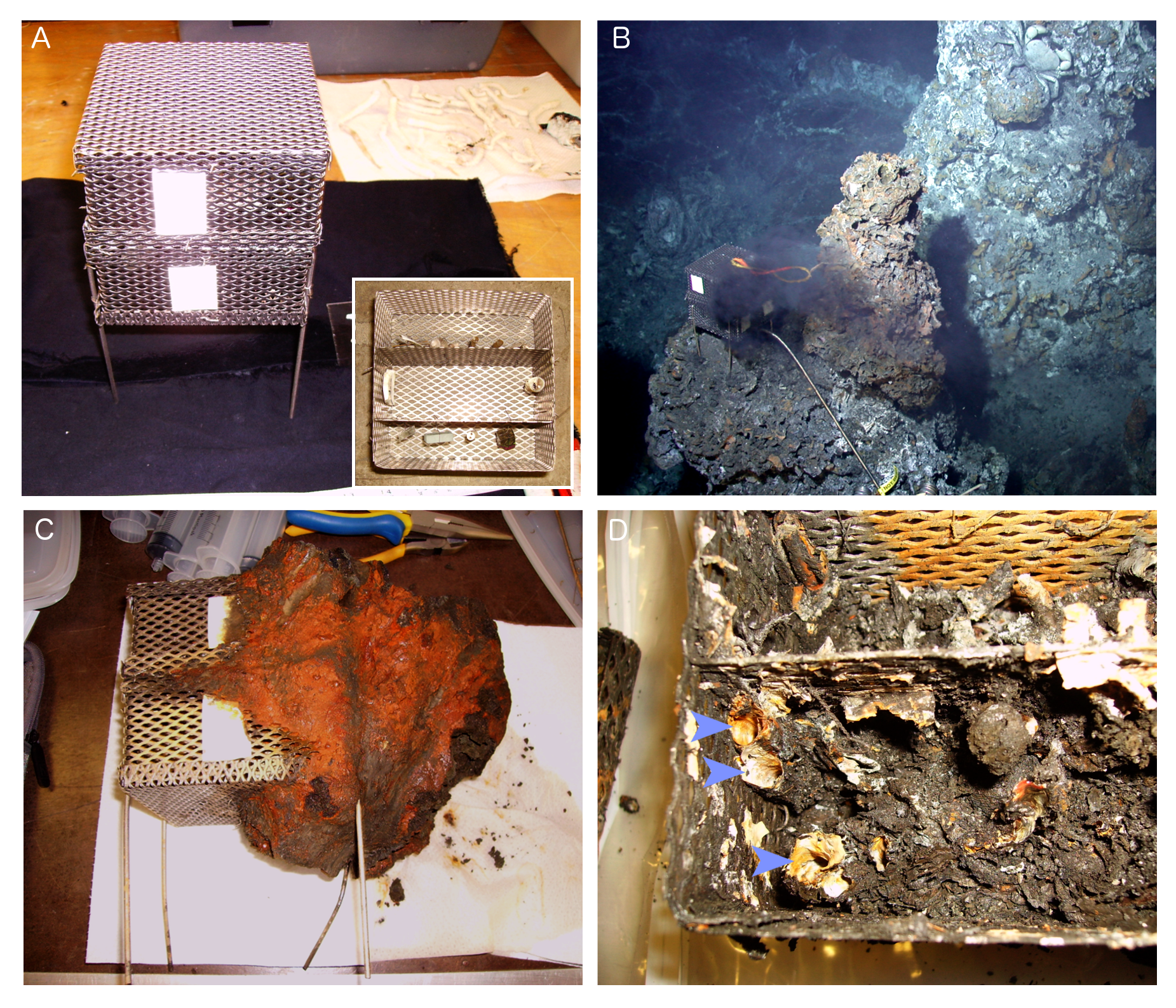

Supplement: Fig S1 — The vent fossilization experiment from which some of the samples used in this study were obtained. [file gbi0013-0152-sd1.tif]

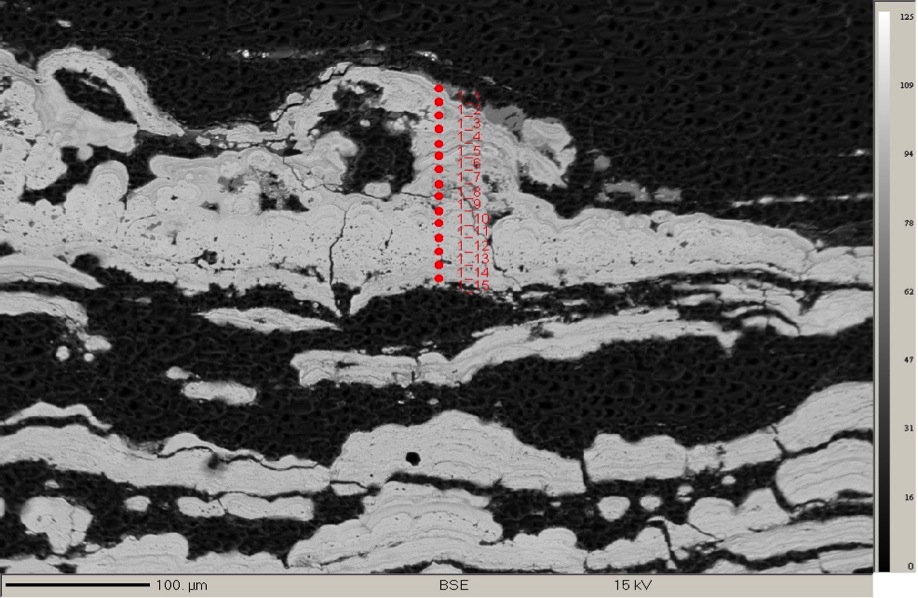

Supplement: Fig S2 — Locations of EPMA point analyses for colloform mineral textures (polished Block 57.1). [file gbi0013-0152-sd2.jpg]

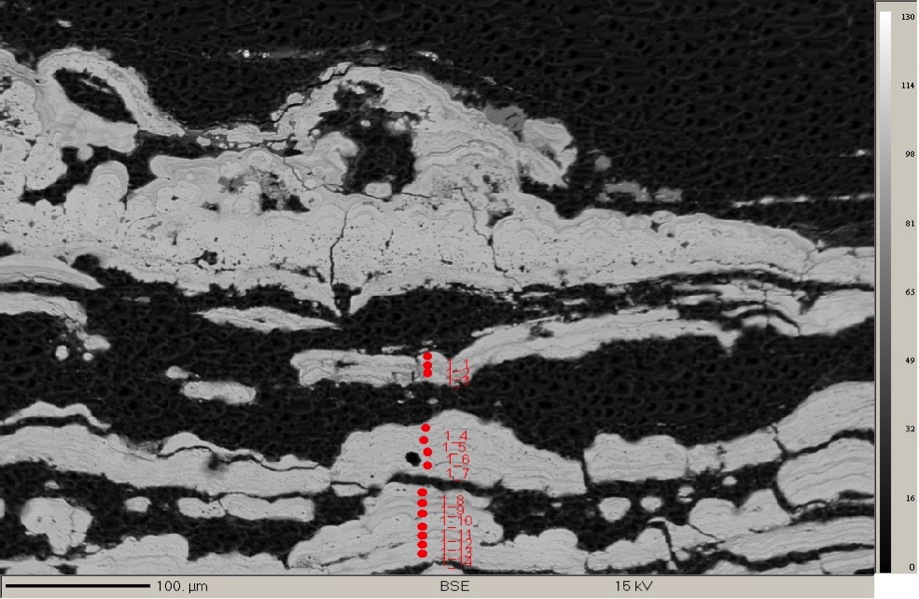

Supplement: Fig S3 — Locations of EPMA point analyses for colloform mineral textures (polished Block 57.1). [file gbi0013-0152-sd3.jpg]

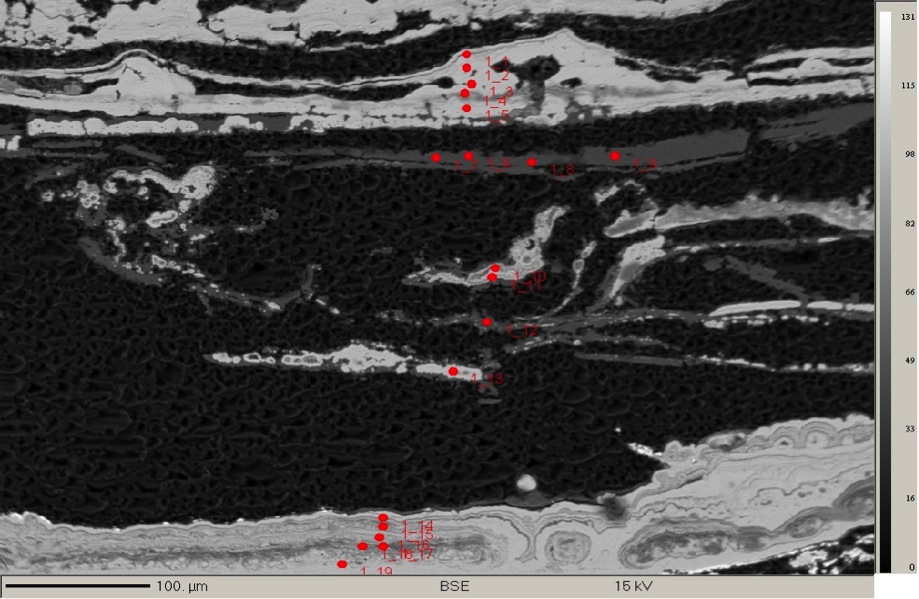

Supplement: Fig S4 — Locations of EPMA point analyses for colloform mineral textures (polished Block 57.1). [file gbi0013-0152-sd4.jpg]

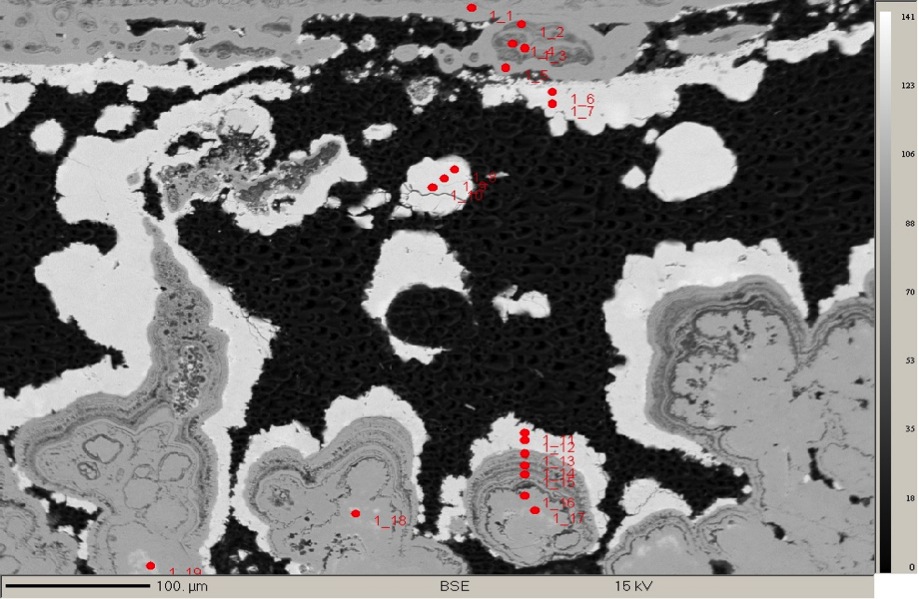

Supplement: Fig S5 — Locations of EPMA point analyses for colloform mineral textures (polished Block 57.1). [file gbi0013-0152-sd5.jpg]
