# Supplementary material for: Mineralization of Alvinella polychaete tubes at hydrothermal vents
Source: Geobiology. 2014 Dec 30;13(2):152–69. doi: 10.1111/gbi.12123 (PMC4359681; doi:10.1111/gbi.12123)
Supplement: Supplementary file 6 — Table S1.Mineralogical observations for each of the polished blocks used in this study. Table S2.EPMA point analyses for colloform mineral textures (polished Block 57.1). Table S3.EPMA point analyses for colloform mineral textures (polished Block 57.1). Table S4.EPMA point analyses for colloform mineral textures (polished Block 57.1). Table S5.EPMA point analyses for colloform mineral textures (polished Block 57.1). Table S6.Information on the diameter measurements of pore and filament associations found within fully mineralized Alvinella spp. tubes, and of microbial filaments from the inner surface of an Alvinella spp. tube (Specimen 44). Methods S1.Fossilization cage experiment. [file gbi0013-0152-sd6.docx]

Table S1. Mineralogical observations for each of the polished blocks used in this study. For type of tube section, T = transverse, L = longitudinal (T, L indicates that both section types are present within the polished block). For mineralization state, P = partially mineralized, F = fully mineralized. For presence of elemental sulphur grains, I = occurring on inner tube surface, O = occurring on outer tube, B = between tube layers, and VCS = within vent chimney sulphide. Element combinations, e.g. FeS, indicate the main elemental constituents of minerals (these are not chemical formulae). ETOH = ethanol.

| Specimen no. | Polished block no. | Type of tube section | From fossilization cages? | Mineralization state | Analysis techniques | No. of organic tube layers | No. of mineral horizons | Description of tube mineralogy | Description of mineralogy directly behind tube | Description of mineralogy of remainder of section (vent chimney sulphides) | Bacterial textures | Elemental sulfur grains |
| --- | --- | --- | --- | --- | --- | --- | --- | --- | --- | --- | --- | --- |
| 44 | 44.1 | T, L | N | P | SEM, EDX, RL | 3 | 2 - 8 | colloform FeS, CuFeS, and mineral fragments on internal and outer organic layer surfaces | - | - | filaments on surface | I, O, B |
| 44 | 44.2 | T | N | P | RL | 1 | 2 - 6 | small colloform pyrite nodules, fine grained marcasite overgrowth | spongy pyrite-marcasite | - | filaments on surface | - |
| 44 | 44.3 | T | N | P | RL | 2 - 3 | 1 - 3 | small colloform pyrite nodules, fine grained marcasite overgrowth | spongy pyrite-marcasite | - | filaments on surface | - |
| 45 | 45.1 | T, L | N | P | SEM, EDX, RL | 3 - 5 | 4 - 8 | mainly organic, colloform deposition of FeS and CuFeS between layers, mineral fragments | - | - | filaments on surface | - |
| 46 | 46.1 | T, L | N | P | SEM, EDX, RL | 4 | 5 - 11 | mainly organic, FeOS layers some with Zn, grains CuZnFeS, ZnFeS, FeS | - | - | - | I, B |
| 47 | 47.1 | T, L | Y | P | SEM, EDX, RL | 2 | 1 - 3 | colloform FeS and CuFeS between tube layers, some with O, FeOCl deposition on outside of tube, CuFeSO and FeS on inside, with acicular FeS and CuFeS | - | - | - | - |
| 47 | 47.2 | T, L | Y | P | SEM, EDX | 2 | 1 - 3 | FeS FeCuS FeCuZnSO grains, FeOCl patches, acicular FeS all in between layers | - | - | - | - |
| 48 | 48.1 | T, L | N | P | SEM, EDX, RL | 4 | 1 - 7 | colloform FeS, and FeSCaO between layers with pores, CaSO grains on inside of tube, ZnFeS, FeS and CuZnFeS growth and CaSO between tube layers | - | - | pores in colloform FeS layers | B |
| 49 | 49.1 | T, L | N | P | SEM, EDX, RL | 3 | 1 - 3 | CaSO on outside, fine ZnFeS growth on inside, colloform FeS between layers with pores | - | - | pores in colloform FeS layers | I, B |
| 54 | 63983 | T | N | P | RL | 4 | 2 - 17 | FeS horizons stemming from thin layer, FeO in organic tube layers | - | - | - | - |
| 55 | 63979 | T | N | F | SEM | _ | 2-6 | colloform bands of FeS, overgrown on inside of tube by CaSO | Mostly FeS with some ZnS | _ | pores and filaments in colloform FeS | - |
| 56 | 56.1 | T | Y | P | SEM, EDX, RL | 5 | 7 | tube layers FeSO with some Ca, colloform FeS between layers | - | - | - | - |
| 57 | 57.1 | T | Y | F | SEM, EDX, XRD, EPMA | - | 13 - 21 | interlayered colloform FeS and SiO | ZnS overgrowth overgrown by PbS, little FeS | fine intergrained FeS SiO and ZnS, giving way to large crystaline ZnFeS | pores in colloform FeS, pores and filaments in distinct layer, clump | - |
| 57 | 57.2 | T, L | Y | F | SEM, EDX | - | 3 - 18 | interlayered colloform FeS and SiO, FeO on innermost tube layer | SiO and pyrite-marcasite crust | small to larger grained crystalline CuFeS | pores in colloform FeS layers |  |
| 57 | 57.3 | T | Y | F | SEM, EDX | - | 6 - 16 | interlayered colloform FeS and SiO | large grained CaSO or ZnS overgrowing FeS and SiO, FeS stands in between other minerals | large grained CaSO or small grained mixture of ZnS, FeS and SiO | pores in colloform FeS, pores and filaments in distinct layers |  |
| 58 | 58.1 | T | N | F | SEM, EDX | - | 1 - 6 | organic tube layers replaced mainly by small spheres of SiO with FeZnS present, some FeSO also | acicular ZnFeS and grains, abundant SiO, some PbS overgrowth | larger grained FeS and ZnFeS overgrown by SiO | - | - |
| 59 | 59.1 | T, L | N | F | SEM, EDX, RL | - | 2 - 4 | thin pyrite layers, FeSO on innermost tube | spongy pyrite-marcasite | - | - | - |
| 59 | 59.2 | T | N | F | SEM, EDX | - | 2 - 4 | thin FeS layers with some SiO, FeSO around FeS | spongy pyrite-marcasite, thin SiO strands | - | - | O |
| 60 | 60.1 | T | Y | F | SEM, EDX, RL | - | 4 - 27 | interlayered colloform pyrite and SiO, some ZnFeS between tube layers | large round-shaped pyrite with SiO in between gaps | FeS overgrown by ZnFeS leading into more ZnFeS | pores and filaments in colloform pyrite layers, clumps around tubes | - |
| 60 | 60.2 | L | Y | F | SEM, EDX | - | 5 | interlayered colloform FeS and SiO | SiO and spongy pyrite-marcasite, possible tube overgrown by minerals | FeS overgrown by ZnFeS, leading into intergrown FeS with SiO spheres and ZnFeS overgrowth | pores in colloform FeS layers | - |
| 60 | 60.3 | L | Y | F | SEM, EDX | - | 15-57 | highly colloform FeS layers | sheet SiO and FeS strands with ZnFeS overgrowth | large grained ZnFeS overgrowing smaller FeS, fine SiO also present | pores in colloform FeS layers | O |
| 61 | 61.1 | T | Y | F | SEM, EDX | - | 3 - 4 | thin FeS layers | spongy FeS, some ZnFeS overgrowth | spongy FeS, some ZnFeS overgrowth | pores in colloform FeS layers | - |
| 61 | 61.2 | T, L | Y | F | SEM, EDX | - | 3 - 6 | colloform bands of FeS | spongy pyrite-marcasite | acicular and granular FeS, small ZnS grains | pores in colloform FeS inner tube layers, clump colony | - |
| 62 | 62.1 | T, L | N | F | SEM, EDX, RL | - | 9 | thick FeSO layers interlayered with SiO, SiO web-like | spongy pyrite-marcasite, FeS strands and growth next to SiO, strands and spheres of SiO | spongy pyrite-marcasite, some FeS growth onto SiO, strands and spheres of SiO | filaments in round pyrite grains | - |
| 62 | 62.2 | T, L | N | F | SEM, EDX | - | 1 - 3 | inner FeS layers only, inner bands overgrown by more crystalline FeS | - | - | pore in distinct inner tube layers | - |
| 62 | 62.4 | T, L | N | F | SEM, EDX | - | 5 - 7 | FeS stemming from some very thin bands, interlayering with SiO in some places | SiO and round pyrite-marcasite mixture | - | pores not very distinct | - |
| 66 | 63976 | T | N | F | RL | - | 1 - 4 | pyrite tube layers | pyrite overgrown by marcasite | pyrite overgrown by marcasite and ZnS, grains of chalcopyrite | - | - |
| 67 | 63978 | T | N | F | SEM | - | 6-8 | colloform bands of FeS, overgrown on inside of tube by CaSO | FeS then FeS overgrown by ZnS, CaSO fills some gaps | FeS then mainly ZnS with some CuFeS inclusions | pores in colloform FeS layers | - |
| 68 | 63984 | T | N | F | RL | - | 2 - 3 | fine-grained marcasite bands | mainly marcasite, spongy, with size of crystals increasing away from cores | intermixed fine and large grained ZnS, pyrite and chalocpyrite | - | - |
| 69 | 63977 | T | N | F | RL | - | 2 - 4 | thin pyrite bands overgrown by marcasite | intermixed small and large grained marcasite, some acicular | intermixed pyrite, ZnS and marcasite but less marcasite | - | - |
| 70 | 70.1 | T | Y | F | SEM, EDX, RL, EPMA | - | 4 - 6 | highly colloform pyrite or thin FeS bands, inside of tube overgrown by crystalline marcasite and ZnS | ZnS sometimes directly behind tube, some empty space, very porous FeS | mainly porous FeS with some ZnS | pores in colloform FeS layers | - |
| 71 | 71.1 | T | N | F | SEM, EDX | - | 3 - 5 | colloform bands of FeS make up thin tube rim | spongy pyrite-marcasite | fine grained FeS and ZnS with SiO, larger grained ZnS overgrowing FeS | pores in colloform FeS inner tube layers |  |
| 71 | 71.2 | L | N | F | SEM, EDX, RL | - | 4 - 7 | colloform bands of FeS | spongy pyrite-marcasite, some overgrowth by thin band of ZnS with PbS | FeS overgrown by ZnS | pores in colloform FeS inner tube layers | B |
| 71 | 71.3 | T | N | F | SEM, EDX | - | 7 - 9 | bands with colloform FeS and more crystalline FeS overgrowth | spongy FeS | FeS and CuFeS in CaSO, then CuFeS | pores in colloform FeS inner tube layers | - |
| 72 | 72.1 | T | Y | F | SEM, EDX | - | 1 - 3 | colloform bands of FeS | spongy FeS, overgrown by ZnS and FeO | FeS, needle-shaped FeO | pores and filaments in colloform FeS layers | - |
| 72 | 72.2 | T | Y | F | SEM, EDX | - | 4 - 6 | colloform bands of FeS, mineral growth from distinct surfaces | spongy FeS, overgrown by ZnS and FeO | larger FeS, extensively overgrown by acicular FeO and smaller-grained ZnS | pores in colloform FeS, pores and filaments in distinct layers | - |
| 74 | 74.1 | T | N | F | SEM, EDX | - | 4 - 8 | interlayered colloform FeS and SiO, FeS cores overgrown by more crystalline FeS | large round-shaped FeS and SiO in between | - | SiO clumps in large round FeS | B |
| 74 | 74.2 | T | N | F | SEM, EDX | - | 7 - 13 | interlayered colloform FeS and SiO, FeS cores overgrown by more crystalline FeS | large round-shaped FeS and SiO in between | FeS growth from cores,overgrowth by ZnS, larger patches of FeS | pores and filaments in colloform FeS | - |
| 74 | 74.3 | L | N | F | SEM, EDX | - | 4 | interlayered FeS and SiO, inner colloform FeS bands overgrown by more crystalline FeS | large round-shaped FeS and SiO in between | - | filaments in round FeS grains | - |
| 75 | 75.1 | T | N | F | SEM, EDX | - | 9 - 18 | interlayered colloform FeS and SiO, thick SiO bands in some places with thin FeS horizons within them | patchy, stringy silica overgrown by ZnS | small grained FeS and SiO oergrown by larger ZnS, tube remnants | pores in colloform FeS | VCS |
| 75 | 75.2 | T, L | N | F | SEM, EDX | - | 9 - 17 | interlayered colloform FeS and SiO | fine grained FeS and patchy, stringy SiO, or FeS overgrown by ZnS behind tube | patches of fine grained FeS and SiO overgrown by large crystalline ZnS | pores in colloform FeS | - |
| 75 | 75.3 | T | N | F | SEM, EDX | - | 4 - 7 | thin colloform FeS bands, SiO behind | very fine and patchy FeS and SiO | colloform FeS, large grained ZnS behind, SiO present in layers | pores in colloform FeS | VCS |

Table S2. EPMA point analyses for colloform mineral textures (polished Block 57.1). Point locations are pictured in Fig. S2.

| Point | Weight % | | | | | | | | |
| --- | --- | --- | --- | --- | --- | --- | --- | --- | --- |
|  | Mg | Si | P | S | Mn | Fe | Cu | Zn | As |
| 1 / 1 . | 0.000 | 0.029 | -0.002 | 2.079 | -0.003 | 1.986 | 0.022 | 0.024 | -0.006 |
| 1 / 2 . | 0.011 | 0.193 | 0.011 | 47.653 | 0.120 | 41.399 | 0.070 | 0.080 | 0.086 |
| 1 / 3 . | 0.015 | 0.234 | 0.042 | 44.363 | 0.116 | 43.203 | 0.017 | 0.100 | 0.018 |
| 1 / 4 . | 0.021 | 0.067 | 0.029 | 42.932 | 0.083 | 38.391 | -0.041 | 0.025 | 0.112 |
| 1 / 5 . | 0.018 | 0.064 | -0.001 | 49.558 | 0.207 | 42.433 | 0.004 | 0.018 | 0.019 |
| 1 / 6 . | 0.010 | 0.091 | 0.006 | 50.263 | 0.054 | 42.135 | 0.017 | 0.024 | 0.019 |
| 1 / 7 . | 0.011 | 0.037 | 0.002 | 51.547 | 0.089 | 41.579 | -0.014 | 0.040 | 0.013 |
| 1 / 8 . | 0.009 | 0.049 | 0.010 | 44.511 | 0.150 | 38.627 | 0.009 | 0.012 | 0.129 |
| 1 / 9 . | 0.015 | 0.280 | 0.034 | 35.967 | 0.127 | 37.664 | 0.012 | 0.156 | 0.217 |
| 1 / 10 . | 0.018 | 0.058 | 0.008 | 47.821 | 0.350 | 40.289 | 0.000 | 0.084 | 0.050 |
| 1 / 11 . | 0.016 | 0.125 | 0.008 | 48.564 | 0.085 | 41.870 | -0.018 | 0.027 | 0.004 |
| 1 / 12 . | 0.014 | 0.027 | 0.004 | 52.516 | 0.081 | 41.786 | 0.011 | 0.023 | 0.011 |
| 1 / 13 . | 0.012 | 0.326 | 0.036 | 44.808 | 0.066 | 37.967 | 0.003 | 0.066 | 0.016 |
| 1 / 14 . | 0.024 | 0.073 | 0.004 | 51.455 | 0.231 | 40.522 | -0.002 | 0.017 | -0.009 |
| 1 / 15 . | 0.015 | 0.105 | 0.009 | 47.695 | 0.382 | 40.155 | 0.009 | 0.062 | 0.010 |
|  | Detection limit % | | | | | | | | |
| 1 / 1 . | 0.008 | 0.009 | 0.011 | 0.020 | 0.024 | 0.034 | 0.037 | 0.045 | 0.018 |
| 1 / 2 . | 0.013 | 0.013 | 0.015 | 0.048 | 0.042 | 0.059 | 0.076 | 0.090 | 0.025 |
| 1 / 3 . | 0.012 | 0.013 | 0.016 | 0.046 | 0.041 | 0.060 | 0.072 | 0.091 | 0.024 |
| 1 / 4 . | 0.013 | 0.013 | 0.014 | 0.045 | 0.042 | 0.055 | 0.074 | 0.088 | 0.026 |
| 1 / 5 . | 0.012 | 0.012 | 0.017 | 0.047 | 0.043 | 0.058 | 0.073 | 0.093 | 0.024 |
| 1 / 6 . | 0.012 | 0.013 | 0.016 | 0.046 | 0.044 | 0.060 | 0.075 | 0.091 | 0.024 |
| 1 / 7 . | 0.012 | 0.013 | 0.017 | 0.049 | 0.042 | 0.060 | 0.073 | 0.088 | 0.025 |
| 1 / 8 . | 0.012 | 0.013 | 0.016 | 0.047 | 0.042 | 0.059 | 0.072 | 0.089 | 0.025 |
| 1 / 9 . | 0.013 | 0.013 | 0.014 | 0.043 | 0.040 | 0.057 | 0.070 | 0.087 | 0.025 |
| 1 / 10 . | 0.013 | 0.013 | 0.015 | 0.047 | 0.043 | 0.059 | 0.072 | 0.088 | 0.026 |
| 1 / 11 . | 0.012 | 0.013 | 0.015 | 0.048 | 0.042 | 0.059 | 0.077 | 0.092 | 0.025 |
| 1 / 12 . | 0.013 | 0.013 | 0.015 | 0.049 | 0.045 | 0.056 | 0.073 | 0.091 | 0.025 |
| 1 / 13 . | 0.012 | 0.012 | 0.015 | 0.045 | 0.043 | 0.060 | 0.072 | 0.087 | 0.024 |
| 1 / 14 . | 0.012 | 0.013 | 0.016 | 0.048 | 0.042 | 0.060 | 0.072 | 0.091 | 0.025 |
| 1 / 15 . | 0.012 | 0.013 | 0.015 | 0.048 | 0.041 | 0.059 | 0.072 | 0.090 | 0.025 |

Table S3. EPMA point analyses for colloform mineral textures (polished Block 57.1). Point locations are pictured in Fig. S3.

| Point | Weight % | | | | | | | | |
| --- | --- | --- | --- | --- | --- | --- | --- | --- | --- |
|  | Mg | Si | P | S | Mn | Fe | Cu | Zn | As |
| 1 / 1 . | 0.029 | 0.032 | 0.003 | 51.051 | 0.319 | 41.101 | -0.006 | 0.033 | 0.026 |
| 1 / 2 . | 0.016 | 0.217 | 0.029 | 42.511 | 0.365 | 39.611 | 0.008 | 0.094 | 0.145 |
| 1 / 3 . | 0.008 | 0.168 | 0.016 | 34.354 | 0.234 | 36.780 | 0.020 | 0.135 | 0.432 |
| 1 / 4 . | 0.023 | 0.064 | 0.005 | 51.192 | 0.372 | 41.203 | 0.002 | 0.045 | 0.025 |
| 1 / 5 . | 0.014 | 0.237 | 0.009 | 51.405 | 0.351 | 40.717 | -0.018 | 0.097 | 0.006 |
| 1 / 6 . | 0.002 | 0.281 | 0.035 | 3.670 | 0.044 | 7.067 | 0.008 | 0.084 | 0.004 |
| 1 / 7 . | 0.020 | 0.099 | 0.011 | 51.518 | 0.344 | 40.981 | -0.050 | 0.042 | 0.013 |
| 1 / 8 . | 0.013 | 0.167 | 0.006 | 50.146 | 0.320 | 41.635 | 0.035 | 0.094 | 0.015 |
| 1 / 9 . | 0.012 | 0.048 | 0.005 | 51.874 | 0.346 | 40.169 | 0.004 | 0.089 | 0.011 |
| 1 / 10 . | 0.011 | 0.059 | 0.004 | 50.707 | 0.449 | 40.988 | -0.009 | 0.136 | 0.019 |
| 1 / 11 . | 0.017 | 0.032 | 0.002 | 53.186 | 0.444 | 41.383 | -0.002 | -0.014 | -0.010 |
| 1 / 12 . | 0.017 | 0.061 | 0.000 | 51.389 | 0.328 | 40.755 | -0.017 | 0.017 | 0.008 |
| 1 / 13 . | 0.011 | 0.081 | 0.005 | 52.136 | 0.327 | 40.830 | 0.001 | 0.016 | 0.070 |
| 1 / 14 . | 0.016 | 0.457 | 0.007 | 50.772 | 0.362 | 39.280 | -0.019 | 0.047 | 0.080 |
|  | Detection limit % | | | | | | | | |
| 1 / 1 . | 0.012 | 0.012 | 0.016 | 0.048 | 0.044 | 0.060 | 0.077 | 0.093 | 0.025 |
| 1 / 2 . | 0.012 | 0.013 | 0.014 | 0.046 | 0.043 | 0.056 | 0.073 | 0.089 | 0.025 |
| 1 / 3 . | 0.013 | 0.012 | 0.016 | 0.044 | 0.042 | 0.056 | 0.070 | 0.085 | 0.023 |
| 1 / 4 . | 0.012 | 0.013 | 0.015 | 0.048 | 0.044 | 0.057 | 0.074 | 0.091 | 0.026 |
| 1 / 5 . | 0.012 | 0.013 | 0.015 | 0.047 | 0.043 | 0.058 | 0.076 | 0.088 | 0.025 |
| 1 / 6 . | 0.005 | 0.005 | 0.010 | 0.019 | 0.029 | 0.039 | 0.047 | 0.057 | 0.010 |
| 1 / 7 . | 0.012 | 0.013 | 0.014 | 0.047 | 0.045 | 0.062 | 0.076 | 0.093 | 0.025 |
| 1 / 8 . | 0.013 | 0.013 | 0.015 | 0.047 | 0.043 | 0.061 | 0.074 | 0.090 | 0.025 |
| 1 / 9 . | 0.012 | 0.013 | 0.015 | 0.048 | 0.041 | 0.061 | 0.076 | 0.091 | 0.025 |
| 1 / 10 . | 0.013 | 0.013 | 0.016 | 0.044 | 0.043 | 0.064 | 0.074 | 0.087 | 0.024 |
| 1 / 11 . | 0.012 | 0.013 | 0.016 | 0.048 | 0.045 | 0.063 | 0.075 | 0.094 | 0.025 |
| 1 / 12 . | 0.012 | 0.013 | 0.015 | 0.049 | 0.044 | 0.059 | 0.077 | 0.092 | 0.025 |
| 1 / 13 . | 0.012 | 0.013 | 0.016 | 0.048 | 0.040 | 0.059 | 0.075 | 0.093 | 0.025 |
| 1 / 14 . | 0.013 | 0.013 | 0.016 | 0.047 | 0.043 | 0.061 | 0.075 | 0.090 | 0.025 |

Table S4. EPMA point analyses for colloform mineral textures (polished Block 57.1). Point locations are pictured in Fig. S4.

| Point | Weight % | | | | | | | | |
| --- | --- | --- | --- | --- | --- | --- | --- | --- | --- |
|  | Mg | Si | P | S | Mn | Fe | Cu | Zn | As |
| 1 / 1 . | -0.002 | 0.380 | -0.002 | 2.177 | 0.008 | 1.007 | -0.027 | 0.000 | 0.028 |
| 1 / 2 . | 0.008 | 1.399 | 0.001 | 48.325 | 0.282 | 39.618 | -0.030 | 0.052 | 0.022 |
| 1 / 3 . | 0.004 | 0.998 | 0.007 | 48.592 | 0.042 | 42.504 | 0.007 | 0.022 | 0.032 |
| 1 / 4 . | 0.001 | 0.365 | 0.004 | 52.166 | 0.124 | 43.029 | 0.011 | 0.008 | 0.007 |
| 1 / 5 . | 0.018 | 2.119 | 0.002 | 46.179 | 0.106 | 38.310 | -0.005 | 0.080 | 0.086 |
| 1 / 6 . | -0.002 | 1.014 | -0.003 | 0.797 | 0.008 | 0.117 | 0.000 | -0.017 | 0.005 |
| 1 / 7 . | -0.001 | 4.530 | 0.010 | 0.685 | -0.006 | 0.157 | -0.003 | -0.003 | 0.026 |
| 1 / 8 . | -0.001 | 42.249 | 0.005 | 0.092 | -0.012 | 0.207 | -0.003 | -0.001 | 0.004 |
| 1 / 9 . | 0.004 | 46.634 | 0.003 | 0.068 | 0.003 | 0.229 | -0.016 | -0.027 | 0.008 |
| 1 / 10 . | -0.005 | 19.162 | 0.004 | 29.734 | 0.059 | 18.176 | 0.056 | -0.008 | 0.373 |
| 1 / 11 . | 0.000 | 5.239 | 0.005 | 45.927 | 0.024 | 34.594 | 0.036 | 0.028 | 0.119 |
| 1 / 12 . | -0.005 | 39.390 | 0.000 | 2.854 | -0.003 | 1.775 | -0.001 | 0.048 | 0.162 |
| 1 / 13 . | 0.010 | 1.171 | 0.007 | 51.140 | 0.103 | 40.538 | 0.018 | 0.102 | 0.164 |
| 1 / 14 . | 0.008 | 9.973 | 0.005 | 39.117 | 0.050 | 32.143 | 0.011 | 0.127 | 0.044 |
| 1 / 15 . | 0.009 | 6.979 | 0.007 | 44.944 | 0.065 | 34.847 | 0.006 | 0.108 | 0.007 |
| 1 / 16 . | 0.001 | 18.109 | 0.009 | 31.709 | 0.114 | 23.409 | -0.028 | 0.090 | 0.045 |
| 1 / 17 . | 0.019 | 8.185 | 0.005 | 39.423 | 0.279 | 32.313 | -0.014 | 0.152 | 0.081 |
| 1 / 18 . | 0.016 | 4.056 | 0.002 | 46.871 | 0.253 | 37.946 | -0.006 | 0.171 | 0.059 |
| 1 / 19 . | 0.003 | 0.040 | 0.004 | 53.749 | 0.100 | 43.360 | 0.021 | 0.226 | 0.010 |
|  | Detection limit % | | | | | | | | |
| 1 / 1 . | 0.008 | 0.008 | 0.009 | 0.020 | 0.021 | 0.030 | 0.040 | 0.046 | 0.015 |
| 1 / 2 . | 0.012 | 0.013 | 0.016 | 0.045 | 0.042 | 0.058 | 0.078 | 0.090 | 0.025 |
| 1 / 3 . | 0.012 | 0.012 | 0.017 | 0.048 | 0.044 | 0.060 | 0.078 | 0.093 | 0.024 |
| 1 / 4 . | 0.012 | 0.013 | 0.016 | 0.048 | 0.042 | 0.061 | 0.075 | 0.093 | 0.025 |
| 1 / 5 . | 0.012 | 0.013 | 0.015 | 0.046 | 0.043 | 0.060 | 0.074 | 0.091 | 0.025 |
| 1 / 6 . | 0.006 | 0.007 | 0.013 | 0.020 | 0.022 | 0.029 | 0.039 | 0.048 | 0.011 |
| 1 / 7 . | 0.006 | 0.008 | 0.013 | 0.027 | 0.026 | 0.029 | 0.042 | 0.047 | 0.011 |
| 1 / 8 . | 0.006 | 0.016 | 0.018 | 0.031 | 0.034 | 0.041 | 0.056 | 0.068 | 0.013 |
| 1 / 9 . | 0.006 | 0.017 | 0.018 | 0.031 | 0.033 | 0.043 | 0.056 | 0.068 | 0.013 |
| 1 / 10 . | 0.010 | 0.013 | 0.015 | 0.040 | 0.038 | 0.051 | 0.065 | 0.081 | 0.020 |
| 1 / 11 . | 0.012 | 0.013 | 0.016 | 0.046 | 0.044 | 0.061 | 0.070 | 0.090 | 0.023 |
| 1 / 12 . | 0.007 | 0.014 | 0.016 | 0.033 | 0.036 | 0.041 | 0.059 | 0.068 | 0.013 |
| 1 / 13 . | 0.013 | 0.013 | 0.015 | 0.049 | 0.044 | 0.060 | 0.076 | 0.091 | 0.025 |
| 1 / 14 . | 0.011 | 0.013 | 0.016 | 0.044 | 0.041 | 0.057 | 0.069 | 0.088 | 0.023 |
| 1 / 15 . | 0.011 | 0.013 | 0.016 | 0.046 | 0.042 | 0.059 | 0.072 | 0.090 | 0.023 |
| 1 / 16 . | 0.010 | 0.013 | 0.016 | 0.043 | 0.038 | 0.054 | 0.069 | 0.083 | 0.020 |
| 1 / 17 . | 0.012 | 0.013 | 0.016 | 0.044 | 0.042 | 0.056 | 0.070 | 0.087 | 0.023 |
| 1 / 18 . | 0.012 | 0.013 | 0.016 | 0.047 | 0.045 | 0.058 | 0.072 | 0.087 | 0.024 |
| 1 / 19 . | 0.013 | 0.013 | 0.016 | 0.049 | 0.045 | 0.060 | 0.074 | 0.095 | 0.025 |

Table S5. EPMA point analyses for colloform mineral textures (polished Block 57.1). Point locations are pictured in Fig. S5.

| Point | Weight % | | | | | | | | |
| --- | --- | --- | --- | --- | --- | --- | --- | --- | --- |
|  | Mg | Si | P | S | Mn | Fe | Cu | Zn | As |
| 1 / 1 . | 0.018 | 2.241 | 0.014 | 47.715 | 0.243 | 40.174 | -0.039 | 0.137 | 0.077 |
| 1 / 2 . | -0.001 | 0.034 | 0.003 | 52.768 | 0.063 | 44.600 | 0.034 | 0.094 | 0.011 |
| 1 / 3 . | 0.007 | 6.362 | 0.010 | 44.719 | 0.123 | 37.389 | -0.003 | 0.148 | 0.033 |
| 1 / 4 . | 0.010 | 3.234 | -0.002 | 49.817 | 0.150 | 40.659 | 0.040 | 0.134 | 0.020 |
| 1 / 5 . | 0.006 | 14.065 | 0.001 | 37.239 | 0.116 | 29.145 | 0.039 | 0.248 | 0.019 |
| 1 / 6 . | -0.009 | 7.397 | -0.006 | 30.593 | 0.041 | 8.986 | -0.012 | 42.305 | 0.284 |
| 1 / 7 . | -0.024 | 0.004 | -0.007 | 32.428 | 0.001 | 3.407 | 0.011 | 57.248 | 0.251 |
| 1 / 8 . | -0.020 | 0.054 | 0.006 | 31.626 | 0.003 | 2.305 | 0.057 | 54.982 | 0.315 |
| 1 / 9 . | -0.009 | 0.003 | -0.009 | 32.125 | 0.014 | 3.233 | 0.060 | 54.631 | 0.188 |
| 1 / 10 . | -0.010 | 0.006 | -0.008 | 32.070 | 0.015 | 2.952 | 0.032 | 55.486 | 0.186 |
| 1 / 11 . | -0.012 | 0.014 | 0.000 | 32.705 | 0.014 | 5.540 | 0.038 | 55.437 | 0.055 |
| 1 / 12 . | -0.004 | 0.009 | 0.004 | 32.998 | 0.017 | 7.630 | -0.002 | 53.015 | 0.041 |
| 1 / 13 . | 0.012 | 0.120 | 0.012 | 49.151 | 0.055 | 43.113 | -0.010 | 0.797 | 0.021 |
| 1 / 14 . | 0.029 | 5.948 | 0.013 | 42.590 | 0.051 | 37.970 | 0.021 | 0.589 | 0.012 |
| 1 / 15 . | 0.008 | 5.642 | 0.014 | 45.683 | 0.065 | 38.943 | -0.018 | 0.404 | -0.004 |
| 1 / 16 . | 0.005 | 0.007 | 0.006 | 53.999 | 0.038 | 43.159 | -0.023 | 1.985 | 0.038 |
| 1 / 17 . | 0.008 | 0.003 | -0.003 | 54.027 | 0.023 | 43.045 | 0.003 | 1.939 | 0.021 |
| 1 / 18 . | -0.003 | 0.003 | 0.001 | 54.781 | 0.022 | 44.284 | 0.009 | 0.244 | 0.040 |
| 1 / 19 . | 0.007 | 0.004 | -0.006 | 54.517 | 0.077 | 44.312 | 0.013 | 0.274 | 0.013 |
|  | Detection limit % | | | | | | | | |
| 1 / 1 . | 0.012 | 0.013 | 0.016 | 0.047 | 0.042 | 0.059 | 0.078 | 0.093 | 0.024 |
| 1 / 2 . | 0.013 | 0.013 | 0.016 | 0.048 | 0.044 | 0.058 | 0.076 | 0.093 | 0.026 |
| 1 / 3 . | 0.012 | 0.013 | 0.014 | 0.047 | 0.041 | 0.058 | 0.074 | 0.092 | 0.023 |
| 1 / 4 . | 0.013 | 0.013 | 0.016 | 0.048 | 0.043 | 0.059 | 0.074 | 0.095 | 0.025 |
| 1 / 5 . | 0.011 | 0.013 | 0.016 | 0.045 | 0.040 | 0.057 | 0.069 | 0.089 | 0.022 |
| 1 / 6 . | 0.016 | 0.015 | 0.019 | 0.045 | 0.040 | 0.048 | 0.075 | 0.110 | 0.029 |
| 1 / 7 . | 0.018 | 0.016 | 0.020 | 0.045 | 0.046 | 0.054 | 0.080 | 0.119 | 0.032 |
| 1 / 8 . | 0.018 | 0.015 | 0.020 | 0.046 | 0.046 | 0.055 | 0.079 | 0.120 | 0.033 |
| 1 / 9 . | 0.017 | 0.016 | 0.020 | 0.045 | 0.047 | 0.053 | 0.079 | 0.118 | 0.033 |
| 1 / 10 . | 0.017 | 0.016 | 0.020 | 0.048 | 0.044 | 0.054 | 0.080 | 0.121 | 0.032 |
| 1 / 11 . | 0.017 | 0.016 | 0.018 | 0.046 | 0.045 | 0.056 | 0.079 | 0.118 | 0.033 |
| 1 / 12 . | 0.017 | 0.016 | 0.018 | 0.044 | 0.045 | 0.055 | 0.080 | 0.117 | 0.032 |
| 1 / 13 . | 0.013 | 0.013 | 0.015 | 0.047 | 0.043 | 0.058 | 0.076 | 0.095 | 0.026 |
| 1 / 14 . | 0.012 | 0.013 | 0.016 | 0.046 | 0.043 | 0.060 | 0.072 | 0.090 | 0.025 |
| 1 / 15 . | 0.012 | 0.013 | 0.016 | 0.047 | 0.043 | 0.060 | 0.077 | 0.092 | 0.025 |
| 1 / 16 . | 0.013 | 0.013 | 0.016 | 0.050 | 0.044 | 0.063 | 0.079 | 0.094 | 0.025 |
| 1 / 17 . | 0.012 | 0.013 | 0.017 | 0.050 | 0.045 | 0.061 | 0.078 | 0.099 | 0.026 |
| 1 / 18 . | 0.013 | 0.013 | 0.016 | 0.049 | 0.044 | 0.061 | 0.076 | 0.092 | 0.025 |
| 1 / 19 . | 0.013 | 0.013 | 0.016 | 0.048 | 0.045 | 0.064 | 0.077 | 0.091 | 0.025 |

Table S6. Information on the diameter measurements of pore and filament associations found within fully mineralized *Alvinella* spp. tubes, and of microbial filaments from the inner surface of an *Alvinella* spp. tube (Specimen 44). All measurements were taken from SEM images. P/F = pores/filaments.

| Specimen no. | Object measured | Area measured (μm^2^) | No. of pore/filament diameters measured | Pore/filament density (no. per 100 μm^2^) | Maximum diameter (μm) | Minimum diameter (μm) | Average diameter (μm) | Standard deviation (μm) |
| --- | --- | --- | --- | --- | --- | --- | --- | --- |
| 48 | P/F in layers | 766 | 26 | 3.39 | 0.88 | 0.23 | 0.45 | 0.18 |
| 49 | P/F in layers | 3047 | 23 | 0.75 | 1.16 | 0.25 | 0.55 | 0.20 |
| 57 | P/F in layers | 506 | 59 | 11.67 | 0.64 | 0.13 | 0.32 | 0.11 |
| 57 | P/F in layers | 33201 | 66 | 0.20 | 1.24 | 0.21 | 0.49 | 0.21 |
| 59 | P/F in layers | 5230 | 51 | 0.98 | 1.40 | 0.19 | 0.40 | 0.21 |
| 71 | P/F in layers | 3311 | 34 | 1.03 | 2.62 | 0.25 | 0.91 | 0.55 |
| 60 | P/F in clumps | 5905 | 251 | 4.25 | 0.91 | 0.26 | 0.56 | 0.10 |
| 60 | P/F in clumps | 4239 | 104 | 2.45 | 0.96 | 0.36 | 0.61 | 0.11 |
| 74 | P/F in clumps | 2932 | 140 | 4.78 | 0.93 | 0.34 | 0.65 | 0.09 |
| 74 | P/F in clumps | 3495 | 297 | 8.50 | 1.00 | 0.37 | 0.66 | 0.11 |
| 61 | P/F in clumps | 8721 | 147 | 1.69 | 1.36 | 0.47 | 0.79 | 0.16 |
| 44 | Microbial filaments | - | 33 | - | 4.78 | 0.35 | 1.49 | 1.29 |
| 44 | Microbial filaments | - | 62 | - | 3.15 | 0.18 | 1.12 | 0.60 |
| 44 | Microbial filaments | - | 47 | - | 3.65 | 0.24 | 0.96 | 0.67 |

**Methods Supplement**

*Fossilization Cage Experiment*

This experiment involved the deployment of replicate titanium mesh cages approximately 120 mm (length) x 120 mm (width) x 60 mm (height) in size, containing, per cage, invertebrate material (two *Bathymodiolus thermophilus* shell pieces, two gastropod shells, two *Ridgeia piscesae* tube pieces, one shrimp half carapace, one *Calyptogena magnifica* shell piece, and one *Tevnia jerichonana* tube), and a range of abiogenic control materials (Fig. S1A). These cages were placed at two different vent sites (Bio9 during AT15-13; L-vent during AT15-27) in conditions of high-temperature fluid flow, i.e. on top of vent chimneys (two cage stack) (Fig. S1B), and in adjacent areas of diffuse flow (two cage stack). In addition, a single control cage was placed away from hydrothermal fluid activity. The cages were deployed for approximately one year (Table 1), and during this time, sulphides (and other vent minerals) had grown on both the inside and outside of the cages deployed on the tops of chimneys (Fig. S1C). Fully mineralized *Alvinella* spp. tubes were apparent on the exterior surfaces of the sulphides that had grown onto the experimental cages (Fig. S1C). Inside one of the cages deployed in diffuse flow there were many non-mineralized and partially mineralized *Alvinella* spp. tubes (Fig. S1D).
